# The effectiveness of E-health interventions promoting physical activity in cancer survivors: a systematic review and meta-analysis of randomized controlled trials

**DOI:** 10.1007/s00432-023-05546-9

**Published:** 2024-02-02

**Authors:** Kangjiao Xiao, Li Tang, Yingtong Chen, Jiahui Zhou, Qiaolan Yang, Rui Wang

**Affiliations:** 1 School of Nursing, Guangdong Pharmaceutical University, Guangzhou, China; 2 Department of Nursing, Guangdong Second Provincial General Hospital, Guangzhou, China

**Keywords:** E-health, Cancer survivors, Physical activity, Meta-analysis

## Abstract

**Purpose:**

This systematic review and meta-analysis aimed to identify whether E-health interventions effectively improve physical activity (PA) in cancer survivors.

**Methods:**

PubMed, Web of Science, and Cochrane Library databases were searched from inception to October 21, 2023. Randomized controlled trials reporting the effect of E-health interventions on PA among cancer survivors were included. Random-effect models were used to calculate standardized mean differences (SMD) and the 95% confidence interval (CI).

**Results:**

In total, 15 trials with 2,291 cancer survivors were included in this meta-analysis. The results showed that E-health interventions improved moderate to vigorous physical activity (MVPA) among cancer survivors (SMD = 0.26, 95% CI 0.08, 0.43, N = 8, *p* < 0.001, I^2^ = 37%), as well as moderate physical activity (MPA) (SMD = 0.22, 95% CI 0.05, 0.38, N = 9, *p* < 0.001, I^2^ = 28%) and vigorous physical activity (VPA) (SMD = 0.34, 95% CI 0.15, 0.54, N = 6, *p* < 0.001, I^2^ = 11%).

**Conclusion:**

E-health interventions are effective at promoting PA among cancer survivors. As current research primarily focuses on immediate post-intervention measurements with limited follow-up data, further investigation is required to explore the long-term effects of E-health interventions on PA.

**Supplementary Information:**

The online version contains supplementary material available at 10.1007/s00432-023-05546-9.

## Introduction

In 2020, there were an estimated 1930 million new cancer cases and nearly 10 million cancer deaths worldwide (Siegel et al. 2021). Fortunately, with advances in both earlier detection and cancer treatments, the 5-year survival rate for most cancers has increased, leading to a rise in the population of cancer survivors (Allemani et al. 2018). Although treatment efficacy has improved, cancer survivors are often plagued with lingering treatment side effects (Campbell et al. 2019) as well as coping with the risk of cancer recurrence (Luigjes-Huizer et al. 2022) and reduced quality of life (Liska and Kolen 2020).

Physical activity (PA) is defined as any bodily movement produced by skeletal muscles that results in an expenditure of energy (WHO 2022). Engaging in regular PA optimizes health outcomes (Rock et al. 2022), treatment effectiveness (Hojman et al. 2018), tolerance (Hojman et al. 2018), and quality of life (QoL) (Liska and Kolen 2020), while also reducing the risk of mortality and recurrence in cancer survivors (Morishita et al. 2020). Despite the known benefits of PA, adherence to the recommended guidelines (engage in > 10 MET-h/week of PA) is low among cancer survivors (Hyland et al. 2018), with only 7% of them participating in sufficient exercises (Avancini et al. 2020). Numerous studies have shown that behavioral health interventions increase PA levels in cancer survivors (Finne et al. 2018). With the rapid development of electronic technology and the proliferation of smart devices, E-health interventions for cancer survivors have become an important part of non-drug interventions (Wong et al. 2018).

E-health interventions refer to the utilization of electronic devices and the Internet to deliver or enhance health services (Eysenbach 2001). E-health interventions have been verified to be effective in a variety of health behavior promotion, including weight loss (Podina and Fodor 2018), depression and anxiety reduction (Massoudi et al. 2019), and PA promotion (Yu et al. 2023). However, there is a lack of evidence focusing on the effects of E-health interventions on the PA levels of cancer survivors. To our knowledge, only Haberlin et al. have conducted a systematic review to evaluate the effect of E-health interventions on PA among cancer survivors (Haberlin et al. 2018). However, E-health interventions have shown inconsistent results in improving PA in cancer survivors. Possible reasons include population heterogeneity (e.g. type of cancer, demographic information differences) and heterogeneity of interventions (e.g., type of electronic intervention, duration of intervention).

To our knowledge, this meta-analysis is the first to quantitatively investigate the impact of E-health interventions on promoting PA in cancer survivors. The study aimed to identify whether E-health interventions effectively improve various aspects of PA, including total PA (TPA), moderate PA (MPA), moderate to vigorous PA (MVPA), vigorous PA (VPA), light PA (LPA), and step counts among cancer survivors.

## Methods

This systematic review followed the guidelines outlined in the Preferred Reporting Items for Systematic Reviews and Meta-Analyses (PRISMA) statement (Page et al. 2021) and adhered to the methodological recommendations provided in the Cochrane Collaboration Handbook (Cumpston et al. 2019). The review was registered on the PROSPERO platform (registration number: CRD42023409365).

### Search strategy

The search encompassed relevant literature from various databases, including Web of Science, PubMed, and Cochrane Library, with no restrictions on publication time or language. The search period extended from the inception of the databases to October 21, 2023. An exhaustive search using Boolean logical operators was conducted using the title and abstract to ensure comprehensive coverage. The specific search information for each database can be found in Table S1.

Two reviewers (KJ-X and JH-Z) independently screened the titles and abstracts of all imported studies to identify potentially relevant studies that met the inclusion criteria. Subsequently, a thorough review of the full-text articles was conducted independently by the same two reviewers (KJ-X and JH-Z) to select studies that were suitable for inclusion in this review. In cases of disagreement or uncertainty regarding the inclusion of studies, a consensus was reached through consultation with a third reviewer (R-W).

### Study selection

Studies that included adult cancer survivors (18 years or older) who had been discharged following cancer treatment were considered eligible for inclusion. The inclusion criteria for this study were limited to published randomized controlled trials (RCTs), including pilot RCTs. Quasi-experiments, cross-sectional surveys, and other qualitative studies were excluded from consideration. There were no restrictions placed on gender, cancer type, cancer stage, region, or nationality of the participants.

E-health interventions were required to be designed with the goal of enhancing PA. E-health interventions refer to any interventions that incorporate at least one of the following components: smartphone apps, wearable activity trackers, websites, phone calls, video consultation, and electronic messages (e.g., text messages, social media messages, email) (Peng et al. 2022; Zhang et al. 2023). Both single and multi-modal e-health interventions were included. In cases involving multiple group comparisons, we only considered comparisons between multi-modal e-health intervention groups and the control group to capture the maximum effect of the e-health intervention.

Comparators were defined as non-eHealth intervention groups (e.g., face-to-face intervention, usual-care waiting-list control, receipt of printed written information). Control groups that solely relied on devices to measure physical activity or employed E-health for general cancer management without a direct connection to increasing physical activity were not excluded.

Studies that measured PA using self-report questionnaires or electronic monitoring devices such as pedometers or accelerometers were included in the analysis. The PA outcomes of interest included MVPA, MPA, VPA, LPA, and step counts. In cases where multiple measurements of PA were taken at different time points, the PA level measured closest to the post-intervention period was selected for analysis. Articles with PA as a secondary outcome measure are excluded.

### Data extraction

We utilized Microsoft Word to extract the information and data from included studies. Two authors independently reviewed and extracted essential information. The extracted critical information included the following aspects: study characteristics (authors, publication year, country), participant characteristics (age, female ratio, cancer type), intervention details (E-health intervention method, E-health intervention duration, comparison), outcome measure (PA measurement instrument, PA level access time, PA level measures, outcome units) and study design (RCT or pilot RCT, sample size). In case of any disagreements in data extraction, they were resolved through discussion between the two authors and consultation with the corresponding author. For missing data, we contacted the corresponding authors of relevant studies via email to obtain the required information.

### Risk of bias (ROB) and quality assessment

The risk of bias in the included studies was assessed using the Cochrane Collaboration risk of bias tool (Higgins and Green 2011). This tool evaluated the risk of bias across seven domains: (1) random sequence generation, (2) allocation sequence concealment, (3) blinding of participants, (4) blinding of outcome assessment, (5) incomplete outcome data, (6) selective outcome reporting, (7) other potential biases (such as small sample size and conflict of interest). Each domain was evaluated as low, unclear, or high risk of bias for each study. The overall risk of bias for each study was determined based on the combination of these seven domains. A study was classified as high risk of bias if more than one domain was assessed as high risk. If the majority of the domains (over three) were assessed as unclear and no high-risk domains were identified, the study was classified as unclear risk of bias. When there were no high-risk domains or less than three domains assessed as unclear, the study was classified as low risk of bias. The Review Manager software (Revman 5.4; The Cochrane Collaboration, The Nordic Cochrane Centre, Copenhagen, Denmark) was used to generate the risk of bias Figure S1. The assessment of risk of bias was conducted by two reviewers, and any disagreements were resolved through negotiation or consultation with a third author.

### Statistical analyses

Multiple PA level measures (MVPA, MPA, VPA, TPA, LPA, step) were analyzed separately. The mean (M) and standard deviation (SD) of each outcome at baseline and post-intervention were drawn to calculate effect sizes based on Cochrane Collaboration Handbook recommendations (Cumpston et al. 2019). First, the effect sizes were pooled using random effect models to assess the principal impact of E-health intervention. Standardized mean difference (SMD) representing the pooled effect sizes were supplied, along with 95% confidence intervals (CIs). If the mean (M) and 95% confidence interval (CI) were provided, the standard deviation (SD) was calculated using the appropriate formula (Cumpston et al. 2019). In cases where the mean and standard deviation were not provided, we utilized statistical transformation methods or contacted the original authors to obtain the necessary data. Second, subgroup analyses of six moderators conducted in this review are presented as follows: (1) E-health intervention method (mobile-based vs. wed-based vs. mixed), (2) country (European country vs. American country vs. Australia), (4) instrument (electronic monitoring vs. self-reports), (5) E-health intervention duration (> 3 months vs. ≤ 3 months), (6) outcome unit (min/week vs. MET*min/week vs. min/day). Moreover, I^2^ statistics and Cochran’s Q-test were used to determine the statistical heterogeneity. When I^2^ was below 25%, between 25 and 50%, between 50 and 75%, and above 75%, it was classified as very low, moderate, medium, and high heterogeneity, respectively, and *p* < 0.1 for Q test was assessed as statistically significant (Higgins and Thompson 2002). Publication bias was assessed using funnel plots and Egger's test (Sterne et al. 2001), with statistical significance set at a *p* < 0.1. Additionally, a sensitivity analysis was performed to ensure the robustness of the pooled effect size. These analyses involved systematically removing each study from the meta-analysis and re-evaluating the effect sizes to assess their impact on the results.

All data calculations (such as effect size syntheses, subgroup analysis, heterogeneity tests, and sensitivity analysis) were performed using the statistical software R4.2.2 (“meta” package).

## Results

### Literature search

**Fig. 1:**
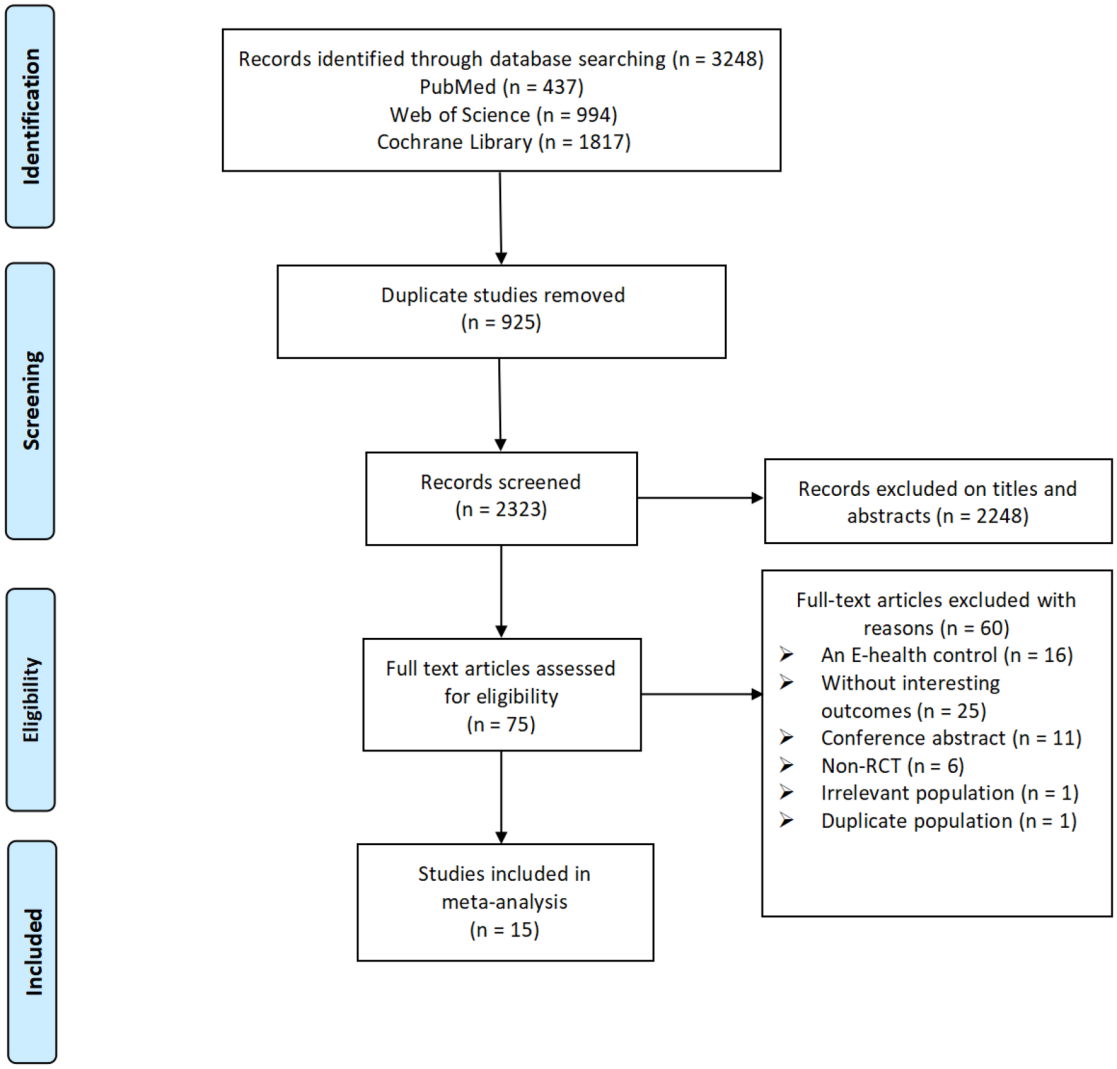
PRISMA Diagram identifying the effects of E-health interventions on physical activity in cancer survivors

The initial search across three electronic databases yielded a total of 3,248 records. After removing 925 duplicate records, the remaining 2,323 records underwent title and abstract screening. Following a careful review of the titles and abstracts, 75 records proceeded to the next step of full-text screening. From this stage, 60 full-text studies were excluded due to various reasons: 16 studies had an E-health control group, 25 studies lacked relevant outcome measures, 11 studies were conference abstracts, 6 studies were non-RCTs, 1 study involved an irrelevant population, and 1 study included a duplicate population. Eventually, a total of 15 studies were included in this systematic review and meta-analysis. The process of literature selection is shown in Fig. 1.

### Studies characteristics

This review included a total of 15 studies (Bantum et al. 2014; Evans et al. 2021; Golsteijn et al. 2018; Hardcastle et al. 2023; Hassoon et al. 2021; Holtdirk et al. 2021; Kanera et al. 2017; Krebs et al. 2017; Lahart et al. 2016; Lynch et al. 2019; Maxwell-Smith et al. 2019; Sheppard et al. 2016; Vallerand et al. 2018; Van Blarigan et al. 2019; van de Wiel et al. 2021). Six studies were conducted in American countries, with 5 studies in the USA (Bantum et al. 2014; Hassoon et al. 2021; Krebs et al. 2017; Sheppard et al. 2016; Van Blarigan et al. 2019) and 1 in Canada (Vallerand et al. 2018). Four studies were conducted in Australia (Evans et al. 2021; Hardcastle et al. 2023; Lynch et al. 2019; Maxwell-Smith et al. 2019), and 4 studies were conducted in European countries, including 3 in the Netherlands (Golsteijn et al. 2018; Kanera et al. 2017; van de Wiel et al. 2021), 1 in Germany (Holtdirk et al. 2021), and 1 in the United Kingdom (Lahart et al. 2016).All the included studies were published in English. These 15 studies were conducted between 2014 and 2023, involving a total of 2,291 individuals. Among the included studies, the E-health intervention group comprised 1,161 participants distributed across the 15 trials. The number of participants in intervention group ranged from 10 to 255, with an average of 77 participants per group in the E-health intervention arm. The control group consisted of 1,130 participants spread across the 15 trials. The sample sizes in the control group varied from 12 to 229 participants, with an average of 7participants per group. The participants' mean age ranged from 49.3 to 70.8 years. Regarding cancer type of survivors, 4 studies specifically focused on breast cancer patients, with an exclusively female population. One study targeted metastatic prostate cancer (mPCa) patients, with an exclusively male population. The remaining 10 studies included a mixed-gender population, with 8 studies focusing on one or a combination of two or more specific cancer types, and 2 studies not specifying the cancer type. Detailed information of the included studies was listed in Table 1.

**Table 1:** Characteristics of including studies evaluating E-health intervention for physical activity level in cancer survivors

Author (year)	Country	Study design	Cancer type	Age (mean ± SD), female ratio (%)	Sample size (IG/CG)	Intervention and duration	Comparison	Instrument of PA, assess time	Outcomes (unit)
Kanera et al. (2017)	Netherlands	RCT	Any cancer	IG: 55.6 ± 11.5, 79.2 CG: 56.2 ± 11.3, 80.5	IG: 231–188-169 CG: 231–221-212	Web program for self-management with behavior change techniques, 6 months	Waitlist control	SQUASH (Questionnaire), baseline-6 month-12 month	MPA (min/week)
Holtdirk et al. (2021)	Germany	RCT	BC	IG: 50.07 ± 8.51, 100 CG:49.80 ± 7.98, 100	IG: 181–141 CG: 182–165	Internet-based CBT intervention, 3 months	Usual-care waiting-list control	IPAQ (Questionnaire), baseline-3 month	TPA (MET*min/week)
Golsteijn et al. (2018)	Netherlands	RCT	PCa, CRC	IG: 66.55 ± 7.07, 14.9 CG: 66.38 ± 8.21, 10.9	IG: 249–234-225 CG: 229–223-218	Computer-tailored PA intervention, 4 months	Usual-care waiting-list control	SQUASH (Questionnaire) and Actigraph GT3X-BT (accelerometer), baseline-3 month -6 month	MVPA (min/week)
Bantum et al. (2014)	USA	RCT	Any cancer	IG: 52.4 ± 11.0, 80.1 CG: 49.3 ± 11.0, 84.1	IG: 176–156 CG: 176–147	A six-week online workshop based on the CDSMP, 6 weeks	Waitlist control	GEQ (Questionnaire), baseline-6 month	1. MVPA (min/week) 2. VPA (min/week) 3. MPA (min/week) 4. LPA (min/week) 5. Stretching (min/week)
Lynch et al. (2019)	Australia	RCT	BC	IG: 61.3 ± 5.9, 100 CG: 61.9 ± 7.0, 100	IG: 43–40 CG: 40–40	Behavioral feedback and goal-setting session + Wearable technology activity monitor + Telephone-delivered behavioral counselling, 12 weeks	Waitlist control	Actigraph® GT3X + accelerometer and activPAL™ accelerometer, baseline-12 week	1. MVPA (min/week) 2. Steps (steps/day)
Maxwell-Smith et al. (2019)	Australia	RCT	CRC, EC	IG: 65.26 ± 7.41, 61.8 CG: 62.88 ± 8.37, 38.2	IG: 34–32 CG: 34–29	Fitbit tracker + Telephone health coaching, 12 weeks	Guideline print materials given + no encouragement	Actigraph Link GT9X accelerometer, baseline-12 week	1. MVPA (min/week) 2. MV10 (min/week) 3. MPA (min/week)
Hardcastle et al. (2023)	Australia	RCT	BC, CRC	IG: 63.7 ± 10.1, 88.4 CG: 62.6 ± 11.8, 81.8	IG: 43–33 CG: 44–41	Fitbit tracker + Telephone health coaching, 12 weeks	Usual care: received exercise booklet	ActiGraph GTX9 accelerometer, baseline-12 week	1. MVPA (min/week) 2. MV10 (min/week) 3. MPA (min/week) 4. LPA (min/week)
Vallerand et al. (2018)	Canada	RCT	Hematologic cancer	IG: 50.1 ± 12.7, 61 CG: 59.3 ± 13.6, 60	IG: 26–26 CG: 25–25	12 weekly telephone counseling sessions, 12 weeks	Self-directed exercise	GLTEQ (Questionnaire), baseline-13 week	1. Exercise (min/week)) 2. VPA (min/week) 3. MPA (min/week) 4. LPA (min/week)
Lahart et al. (2016)	UK	RCT	BC	IG: 52.4 ± 10.3, 100 CG: 54.7 ± 8.3, 100	IG: 40–37 CG: 40–33	Telephone support + PA pack with information booklet and DVD, 6 months	Usual-care waiting-list control	IPAQ (Questionnaire), baseline-6 month	1. TPA (MET*min/week) 2. Work-based PA (MET*min/week) 3. Active transport PA (MET*min/week) 4. Domestic PA (MET*min/week) 5. Leisure PA (MET*min/week) 6. Walking PA (MET*min/week) 7. MPA (MET*min/week) 8. VPA (MET*min/week)
Van Blarigan et al. (2019)	USA	RCT	CC, RC	IG: 56 ± 12, 60 CG: 54 ± 11, 57	IG: 21–20 CG: 21–19	A Fitbit Flex™ to track physical activity + Daily text messages, 12 weeks	Received print educational materials and mailed Fitbit Flex™ after 12-week follow-up	Actigraph GTX3 + accelerometers, baseline-12 week	1. MVPA (min/day) 2. MPA (min/day) 3. VPA (min/day) 4. Steps (steps/day)
Sheppard et al. (2016)	USA	RCT	BC	54.7 ± 9.8, 100	IG: 15–10 CG: 16–12	Six individualized phone MI sessions + pedometers, 12 weeks	Usual care	IPAQ-SF (questionnaire), baseline-12 week	1. TPA (min/week) 2. VPA (min/week) 3. MPA (min/week) 4. Walking (min/week) 5. TPA (MET*min/week) 6. VPA (MET*min/week) 7. MPA (MET*min/week) 8. Walking (MET*min/week)
Evans et al. (2021)	Australia	RCT	mPCa	IG: 69.5 ± 6.6, 0 CG: 70.8 ± 10.2, 0	IG: 20–19 CG: 20–19	A computer- tailored website based on behavioral change theories + telE-health consultations, 8 weeks	Waitlist control	The ActiGraph GT3X activity monitor + GLTEQ (Questionnaire), baseline-9 week	1. MVPA (min/day) 2. Steps (steps/day) 3. LPA (min/week) 4. MPA (min/week) 5. VPA (min/week) 6. Resistance training frequency (sessions/week) 7. Resistance training duration (min)
Krebs et al. (2017)	USA	RCT	BC, PCa	59.8 ± 11.4, 96	IG: 44–32 CG: 42–36	Intervention provided on DVD guided by SCT, 3 months	Standard care: health assessment, advice, and counseling by nurse practitioner	GLTEQ (Questionnaire), baseline-3 month	TPA (MET*min/week)
van de Wiel et al. (2021)	Netherlands	RCT	BC, PCa	IG1: 59.3 ± 11.3, 51 IG2: 59.8 ± 11.7, 48 CG: 59.2 ± 14.4, 48	IG1: 45–31 IG2: 46–32 CG: 46–37	IG1: Received tailored PA intervention through IPAS IG2: Received tailored PA intervention through IPAS + monthly phone calls, 6 months	Usual care + printed leaflet on PA guidelines	Actigraph GT3X + activity monitor, baseline-6 month	MVPA (min/week)
Hassoon et al. (2021)	USA	RCT	BC, PC, CC, LC, CxCa, OC, MC	IG1: 64.1 ± 7.2, 79 IG2: 58.1 ± 11.8, 93 CG: 63.9 ± 9.3, 100	IG1: 14–14 IG2: 14–14 CG: 14–14	IG1: MyCoach (AI coaching delivered by smart speaker) IG2: SmartText (autonomous text messaging) compared to control, 4 weeks	Received printed written information	Fitbit Charge HR2 (wearable sensor), baseline-4 week	Steps (steps/day)

*IG* intervention Group, *CG* control group, *RCT* randomized controlled trial, *SQUASH* short questionnaire to assess health enhancing physical activity, *PA* physical activity, *BC* breast cancer, *IPAQ* International Physical Activity Questionnaire, *TPA* total physical activity, *CBT* cognitive behavioral therapy, *MET* metabolic equivalent task, *PCa* prostate cancer, *CRC* colorectal cancer, *MVPA* moderate-to-vigorous physical activity, *MPA* moderate physical activity, *VPA* vigorous physical activity, *LPA* light physical activity, *USA* the United States of America, *UK* the United Kingdom, *GEQ* the Godin Exercise Questionnaire, *CDSMP* the Chronic Disease Self-Management Program, *EC* Endometrial Cancer, *RC* rectal cancer, *MV* moderate-vigorous, *SMS* short message service, *GLTEQ* Godin Leisure-Time Exercise Questionnaire, *7-Day PAR* 7-day physical activity recall, *DVD* digital video disc, *IPAQ-SF* the International Physical Activity Questionnaire Short Form, *MI* motivational interviewing, *mPCa* metastatic prostate cancer, *CC* colon cancer, *LC* lung cancer, *CxCa* cervical cancer, *OC* oral cancer, *MC* melanoma cancer, *AI* artificial intelligence, *NA* not available, *PAR-Q* Physical Activity Readiness Questionnaire, *SCT* social cognitive theory, *IPAS* internet-based PA support program

The E-health interventions in this study were classified into three modalities. Specifically, nine interventions were categorized as mobile-based interventions, involving the use of wearable technology activity monitors, telephone-delivered behavioral counseling, DVDs, and daily text messages. These interventions were implemented either individually or in combination. The second category comprised four web-based interventions, encompassing a web program for self-management with behavior change techniques, an internet-based cognitive-behavioral therapy (CBT) intervention, a computer-tailored PA intervention, and an online workshop based on specific theories. Importantly, it should be noted that certain studies employed a combination of both mobile-based and web-based interventions, indicating that these modalities were not mutually exclusive. Two interventions were classified as mixed interventions. One study utilized a computer-tailored website based on behavioral change theories and E-health consultations, incorporating the use of mobile devices for feedback or consultation. Another study received a tailored PA intervention through an Interactive Physical Activity System (IPAS) and monthly phone calls. The adoption of E-health strategies varied across the included studies, with earlier studies primarily focusing on mobile-based interventions, while later studies incorporated web-based interventions or a combination of both modalities. Among the included studies, 11 studies had a E-health intervention duration of less than three months, whereas 4 studies had a duration exceeding three months. The E-health intervention durations of these studies ranged from 4 weeks to 6 months.

The studies included in this analysis had varying intervention types for control groups. Specifically, among the included studies, four studies utilized a waiting list control group. Three studies employed a waitlist control group, where participants were placed on a waiting list and received usual care during the waiting period. One study provided guideline print materials without any encouragement, 1 studies implemented usual care, 1 study focused on self-directed exercise, 1 study distributed print educational materials and a Fitbit Flex™ after a 12-week follow-up, 1 study followed standard care, 1 study combined usual care with a printed leaflet on PA guidelines, and 1 study provided participants with printed written information.

Out of the included studies, self-report questionnaires were utilized in 7 studies, while electronic monitoring methods were implemented in 6 studies. Furthermore, 2 studies employed a combination of self-report questionnaires and electronic monitoring methods to evaluate the participants’ PA levels. The majority of the studies utilized PA time as a measure to quantify the amount of PA. Additionally, other methods such as step count and MET (Metabolic Equivalent of Task) were employed to assess and measure physical activity levels.

### Quality of included studies

Among the studies included in this analysis, 10 studies were assessed to have a low risk of bias (Bantum et al. 2014; Evans et al. 2021; Golsteijn et al. 2018; Hardcastle et al. 2023; Hassoon et al. 2021; Kanera et al. 2017; Lahart et al. 2016; Maxwell-Smith et al. 2019; Van Blarigan et al. 2019; van de Wiel et al. 2021), indicating a high level of methodological quality and reliability. One study was assessed as having an unclear risk of bias (Holtdirk et al. 2021). Four studies were found to have a high risk of bias (Krebs et al. 2017; Lynch et al. 2019; Sheppard et al. 2016; Vallerand et al. 2018), suggesting potential limitations in their study design or conduct. Specifically, one study exhibited a high risk of bias in terms of random sequence generation, while another study had a high risk of bias in relation to blinding of outcome assessment. Additionally, two studies were identified as having a high risk of bias due to other sources.

### Primary outcomes

**Fig. 2:**
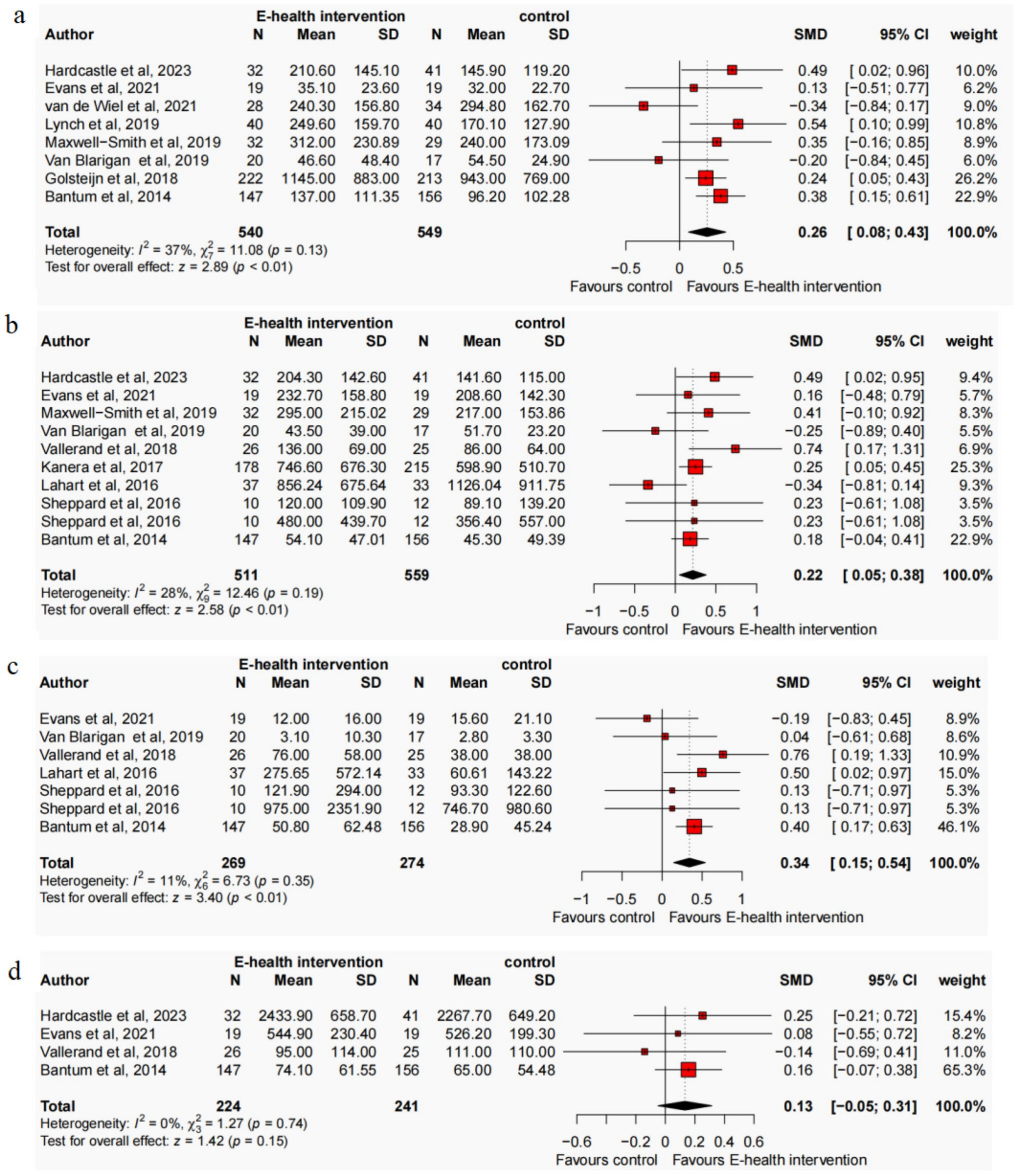
Forest plot of the effect of E-health interventions on MVPA (**a**), MPA (**b**), VPA (**c**), and LPA (**d**) in cancer survivors

**Fig. 3:**
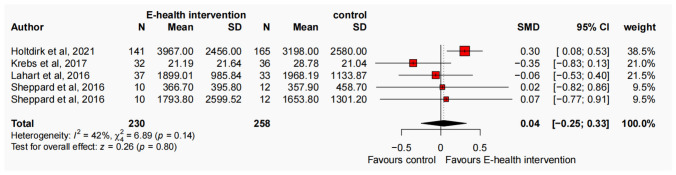
Forest plot of the effect of E-health interventions on TPA in cancer survivors

**Fig. 4:**
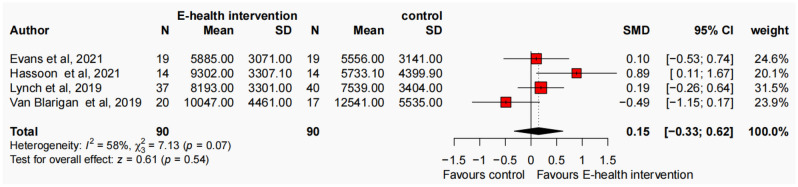
Forest plot of the effect of E-health interventions on step counts in cancer survivors

The present meta-analysis, comprising 15 studies and adopting a random-effects model, investigated the effects of an E-health intervention on PA levels among cancer survivors. At the baseline assessment, the results indicated no significant improvement in MVPA (SMD = − 0.09, 95% CI − 0.29, 0.11, N = 8, *p* = 0.37, I^2^ = 54%), MPA (SMD = − 0.07, 95% CI − 0.27, 0.13, N = 9, *p* = 0.51, I^2^ = 53%), VPA (SMD = 0.01, 95% CI − 0.22, 0.25, N = 6, *p* = 0.91, I^2^ = 34%), LPA (SMD = − 0.21, 95% CI − 0.48, 0.07, N = 4, *p* = 0.14, I^2^ = 43%), TPA (SMD = − 0.02, 95% CI − 0.35, 0.32, N = 4, *p* = 0.93, I^2^ = 58%), and step counts (SMD = − 0.26, 95% CI − 0.61, 0.10, N = 4, *p* = 0.15, I^2^ = 28%) among participants in the E-health intervention group compared to the control group (refer to Figure S2-S7, available in the Supplementary Materials). Following the intervention, significant improvements were observed in MVPA (SMD = 0.26, 95% CI 0.08, 0.43, N = 8, *p* < 0.001, I^2^ = 37%) (Fig. 2a). A total of 1,089 participants were included in the analysis, with individual study sample sizes ranging from 17 to 222. The E-health intervention group comprised 540 participants, while the control group included 549 participants, resulting in an average of 68 participants per group. Additionally, a significant improvement in MPA was observed favours E-health intervention (SMD = 0.22, 95% CI 0.05, 0.38, N = 9, *p* < 0.001, I^2^ = 28%) (Fig. 2b). The analysis included 1,050 participants, with individual study sample sizes ranging from 10 to 215. The E-health intervention group consisted of 501 participants, while the control group included 549 participants, yielding an average of 58 participants per group. Moreover, following the intervention, a significant improvement in VPA was found (SMD = 0.34, 95% CI 0.15, 0.54, N = 6, *p* < 0.001, I^2^ = 11%) (Fig. 2c). The analysis incorporated a total of 523 participants, with individual study sample sizes ranging from 10 to 156. The E-health intervention group included 259 participants, while the control group comprised 264 participants, resulting in an average of 43 participants per group. No significant improvement was found in LPA favours E-health intervention (SMD = 0.13, 95% CI − 0.05, 0.31, N = 4, *p* = 0.15, I^2^ = 0%), TPA (SMD = 0.04, 95% CI − 0.25, 0.33, N = 4, *p* = 0.80, I^2^ = 42%), and step counts (SMD = 0.15, 95% CI − 0.33, 0.17, N = 4, *p* = 0.54, I^2^ = 58%) (refer to Figs. 2d, 3 and 4). To evaluate publication bias, a funnel plot was employed in conjunction with the Egger test, revealing no significant evidence of publication bias at the post-intervention assessment (*p* > 0.1) (refer to Figs. S8–S13, available in the Supplementary Materials).

### Subgroup analysis of PA

Subgroup analyses were conducted to assess the impact of three moderator variables on MVPA, MPA, and VPA at the post-intervention assessment. The instrument used to measure PA levels was the only variable that showed a statistically significant difference, specifically for VPA. VPA measured through self-reports yielded statistically significant results, while VPA measured via electronic monitoring did not demonstrate statistical significance. There were no statistically significant differences observed for MVPA and MPA based on the instrument used for assessment. However, when considering subgroups based on E-health intervention method, duration of E-health intervention, participants from different countries, and outcomes units, no statistically significant differences were found for MVPA, MPA, and VPA. Detailed information on the subgroup analyses and their corresponding results can be found in Figure S19 to S33 in the supplementary materials.

Due to the limited number of included studies (less than 5), no subgroup analyses were conducted to examine the effects of TPA, LPA and step counts.

### Robustness of the results

The sensitivity analyses consistently showed that the effect sizes for MVPA, MPA, and VPA at the post-intervention assessment remained stable and statistically significant (*p* < 0.01). However, it should be noted that the sensitivity analyses revealed that the effect sizes for TPA, LPA, and step counts at the post-intervention assessment were less consistent and did not reach statistical significance (refer to Figure S14-S19, available in the Supplementary Materials).

## Discussion

This study is the first meta-analysis of RCTs designed to assess the effect of E-health interventions on PA in cancer survivors. Across 15 RCTs sampling over 2,291 cancer survivors, primarily mixed cancers (n = 9, 60%), breast cancer (n = 5, 33.3%), and hematologic cancer (n = 1, 6.7%), we found that E-health interventions improved PA levels of cancer survivors.

Specifically, this review demonstrated that E-health interventions effectively improved MVPA, MPA, and VPA among cancer survivors. E-health refers to information and communication technologies (ICT) related health services (Eysenbach 2001). Previous studies have shown that behavior change techniques (BCTs) based on behavior change theories are the core components of ICT (Michaelsen and Esch 2022). A meta-analysis suggests that certain BCTs were associated with increased PA among cancer survivors (Finne et al. 2018). A study involving 68 colorectal and endometrial cancer survivors found a significant effect when adding BCT to E-health interventions (Maxwell-Smith et al. 2019). Furthermore, a recent RCT investigating different approaches to promote PA in 80 metastatic prostate cancer survivors discovered that web- and telephone-based personalized exercise interventions with BCT for metastatic prostate cancer improved PA (Evans et al. 2021). Thus, based on BCT, E-health could be beneficial for enhancing PA among cancer survivors.

The current review showed that E-health interventions did not significantly increase TPA, LPA, or step counts among cancer survivors, this could have been due to the limited number of studies included in the analysis. Despite the limited research on step counts, walking has emerged as the preferred mode of PA among patients with various types of cancer (Elshahat et al. 2021). Tailoring a PA program to the preferences of cancer survivors may have beneficial outcomes for long-term PA maintenance (Wong et al. 2018). Compared with healthy population, cancer survivors are often more suitable for LPA due to their disease progression (Thraen-Borowski et al. 2017). The physical and psychological burdens faced by cancer survivors may often lead to worse adherence to face-to-face interventions. E-health interventions, as a type of intervention mainly mediated by electronic devices and the internet, can better facilitate cancer survivors and increase their adherence.

Research has shown that E-health interventions may have varying levels of effectiveness across different cancer types. For instance, it was observed that computer-tailored interventions were more effective for colorectal cancer participants compared to those with prostate cancer (Golsteijn et al. 2018). E-health interventions predominantly focuses on mixed cancers in included studies. Considering the differences in pathological characteristics of different cancers, it is necessary to conduct intervention trials for specific cancer patients to strengthen our research conclusions. For example, lung cancer is a leading cause of cancer-related mortality (Sung et al. 2021). Patients diagnosed with lung cancer often have a higher burden of symptoms than patients with other prevalent cancers and are less likely to receive support to manage these symptoms (Osowiecka et al. 2023). PA has many benefits for the physical and mental health of those diagnosed with lung cancer (Teba et al. 2022). However, none of the included studies specifically investigated the effects of E-health interventions on PA in lung cancer survivors.

Several studies have confirmed that web-based interventions have a significant promoting effect on the PA of cancer survivors (Golsteijn et al. 2018; Kanera et al. 2017). Mobile-based interventions have also shown a significant promoting effect on PA among cancer survivors (Hassoon et al. 2021). Mobile-based interventions have the potential to offer cancer survivors a flexible, personalized, and convenient way to engage in regular PA (Monteiro-Guerra et al. 2020). Moreover, Evans et al. demonstrated that combining web-based and mobile-based interventions is also effective in promoting PA among cancer survivors (Evans et al. 2021). However, van de Wiel et al. found that online resources combined with telephone counseling interventions were no more effective than online interventions (van de Wiel et al. 2021). Notably, the average age of cancer survivors included in the study was over 59 years old. On one hand, it might be because some cancer survivors, particularly those in the older age group, encountered difficulties when using websites. On the other hand, some patients perceived the telephone calls as a monitoring function rather than a supportive interaction. Our analyses did not show a clear advantage for either E-health intervention, due to the limited number of available studies. The differences in the effects of different types of E-health interventions should be highlighted in future studies.

There is no gold standard for assessing PA among cancer survivors, and two primary methods (electronic monitoring and self-report) were employed across the studies. The self-report group demonstrated larger intervention effects post-intervention compared to the electronic monitoring group (Golsteijn et al. 2018). Self-reporting is an attractive method for data collection due to its low cost, feasibility, and convenience. However, It's important to note that self-report questionnaires could potentially lead to overestimating actual PA levels. Electronic monitoring offers a precise and accurate means of measurement, but it comes with challenges when applied to certain activities (e.g., resistance exercise, cycling, or water-based exercise). Therefore, it is likely that PA level in cancer survivors during these activities was underestimated (Singh et al. 2022).Combining self-report questionnaires with electronic monitoring might present the most complete insight in PA.

## Strengths and limitations

This is the first meta-analysis, to our knowledge, to review the effectiveness of E-health interventions in increasing PA levels among cancer survivors. The low heterogeneity observed in the meta-analysis reinforces the credibility and reliability of these findings, and no publication bias was detected.

Despite the innovation and strength of evidence in this study, there are still several limitations that should be acknowledged. Firstly, the existing research focuses on breast, colorectal, and prostate cancer, which implies that the findings may have limited generalizability to other cancer survivors. Secondly, the nature of E-health interventions made it impossible to blind participants, potentially impacting the quality of risk of bias assessments in the included studies. Thirdly, our review focused solely on immediate PA outcomes following the E-health interventions, lacking information on their long-term effects. Fourthly, the geographical scope of the studies included in our analysis was confined to developed countries. This limitation may restrict the generalizability of our findings to some developing countries. Lastly, despite our comprehensive efforts, there is a possibility that we missed some relevant studies during the literature search, particularly those published in grey literature or not meeting specific search criteria.

## Conclusions

The findings of our review demonstrate that E-health interventions are effective in increasing PA levels among cancer survivors when considering MVPA, MPA, and VPA as outcomes. Our results have clinical and public health implications because they provide support for recommending E-health interventions among cancer survivors as a feasible and scalable population-based strategy to reduce physical inactivity. Nevertheless, further research is needed to optimize E-health interventions to improve usability and adherence to Internet-based PA interventions among cancer survivors. Additionally, balancing research across various cancer types, especially lung cancer, is beneficial for designing tailored E-health interventions for different cancer survivors to improve PA levels. Furthermore, the long-term maintenance of PA improvement through electronic health interventions requires further investigation.

## Supplementary Information

Below is the link to the electronic supplementary material.

Supplementary file1 (DOCX 5066 KB)

## Data Availability

The original contributions presented in the study are included in the article supplementary material; further inquiries can be directed to the corresponding author.
