# Supplementary material for: The effectiveness of E-health interventions promoting physical activity in cancer survivors: a systematic review and meta-analysis of randomized controlled trials
Source: J Cancer Res Clin Oncol. 2024 Feb 2;150(2):72. doi: 10.1007/s00432-023-05546-9 (PMC10837252; doi:10.1007/s00432-023-05546-9)
Supplement: Supplementary file 1 — Supplementary file1 (DOCX 5066 KB) [file 432_2023_5546_MOESM1_ESM.docx]

**Search ran:** 2023.10.21

**Table S1.** Search terms in each database

| **Databases** | **Step** | **Searches** | **Results** |
| --- | --- | --- | --- |
| PubMed | #1 | "cancer"[Title/Abstract] OR "cancers"[Title/Abstract] OR "tumor"[Title/Abstract] OR "tumors"[Title/Abstract] OR "neoplasia"[Title/Abstract] OR "neoplasias"[Title/Abstract] OR "malignancy"[Title/Abstract] OR "malignancies"[Title/Abstract] OR "neoplasm"[Title/Abstract] OR "neoplasms"[Title/Abstract] OR "carcinoma"[Title/Abstract] OR "carcinomas"[Title/Abstract] | 3,775,358 |
|  | #2 | "web"[Title/Abstract] OR "internet"[Title/Abstract] OR "text messaging"[Title/Abstract] OR "digital health"[Title/Abstract] OR "eHealth"[Title/Abstract] OR "e-health"[Title/Abstract] OR "smartphone"[Title/Abstract] OR "phone"[Title/Abstract] OR "mobile device"[Title/Abstract] OR "technology"[Title/Abstract] OR "mobile health"[Title/Abstract] OR "mHealth"[Title/Abstract] OR "m-health"[Title/Abstract] OR "online"[Title/Abstract] OR "website"[Title/Abstract] OR "app"[Title/Abstract] OR "telephone counseling"[Title/Abstract] OR "e-counseling"[Title/Abstract] OR "computer"[Title/Abstract] OR "email"[Title/Abstract] | 1,234,502 |
|  | #3 | "physical activity"[Title/Abstract] OR "physical activities"[Title/Abstract] OR "physical training"[Title/Abstract] OR "exercise"[Title/Abstract] OR "exercises"[Title/Abstract] OR "exercising"[Title/Abstract] OR "sport"[Title/Abstract] OR "sports"[Title/Abstract] OR "step"[Title/Abstract] OR "steps"[Title/Abstract] OR "bicycle"[Title/Abstract] OR "bicycling"[Title/Abstract] OR "bike"[Title/Abstract] OR "biking"[Title/Abstract] OR "walk"[Title/Abstract] OR "walking"[Title/Abstract] OR "run"[Title/Abstract] OR "running"[Title/Abstract] OR "jog"[Title/Abstract] OR "jogging"[Title/Abstract] OR "swim"[Title/Abstract] OR "swimming"[Title/Abstract] OR "dance"[Title/Abstract] OR "dancing"[Title/Abstract] OR "plyometrics"[Title/Abstract] OR "resistance training"[Title/Abstract] | 1,537,832 |
|  | #4 | "RCT"[Title/Abstract] OR "randomized clinical trial"[Title/Abstract] OR "randomized controlled trial"[Title/Abstract] OR "randomized trial"[Title/Abstract] | 220,936 |
|  | #5 | #1 AND #2 AND #3 AND #4 | 437 |
| Web of Science | #1 | **TI=(cancer OR cancers OR tumor OR tumors OR neoplasia OR neoplasias OR malignancy OR malignancies OR neoplasm OR neoplasms OR carcinoma OR carcinomas)** | 2,211,300 |
| **Databases** | **Step** | **Searches** | **Results** |
|  | #2 | **TI=(web OR internet OR text messaging OR digital health OR eHealth OR e-health OR smartphone OR phone OR mobile device OR technology OR mobile health OR mHealth OR m-health OR online OR website OR app OR telephone counseling OR e-counseling OR computer OR email)** | 449,398 |
|  | #3 | **TI=(physical activity OR physical activities OR physical training OR exercise OR exercises OR exercising OR sport OR sports OR step OR steps OR bicycle OR bicycling OR bike OR biking OR walk OR walking OR run OR running OR jog OR jogging OR swim OR swimming OR dance OR dancing OR plyometrics OR resistance training)** | 429,043 |
|  | #4 | **TI=(RCT OR randomized clinical trial OR randomized controlled trial OR randomized trial)** | 239,426 |
|  | #5 | #1 AND #2 AND #3 AND #4 | 54 |
|  | #6 | AB=(cancer OR cancers OR tumor OR tumors OR neoplasia OR neoplasias OR malignancy OR malignancies OR neoplasm OR neoplasms OR carcinoma OR carcinomas) | 2,487,725 |
|  | #7 | AB=(web OR internet OR text messaging OR digital health OR eHealth OR e-health OR smartphone OR phone OR mobile device OR technology OR mobile health OR mHealth OR m-health OR online OR website OR app OR telephone counseling OR e-counseling OR computer OR email) | 2,029,678 |
|  | #8 | AB=(physical activity OR physical activities OR physical training OR exercise OR exercises OR exercising OR sport OR sports OR step OR steps OR bicycle OR bicycling OR bike OR biking OR walk OR walking OR run OR running OR jog OR jogging OR swim OR swimming OR dance OR dancing OR plyometrics OR resistance training) | 2,101,280 |
|  | #9 | AB=(RCT OR randomized clinical trial OR randomized controlled trial OR randomized trial) | 394,748 |
|  | #10 | #6 AND #7 AND #8 AND #9 | 722 |
|  | #11 | #5 OR #10 | 743 |
| Cochrane Library | #1 | (cancer OR cancers OR tumor OR tumors OR neoplasia OR neoplasias OR malignancy OR malignancies OR neoplasm OR neoplasms OR carcinoma OR carcinomas):ti | 159,311 |
|  | #2 | (web OR internet OR text messaging OR digital health OR eHealth OR e-health OR smartphone OR phone OR mobile device OR technology OR mobile health OR mHealth OR m-health OR online OR website OR app OR telephone counseling OR e-counseling OR computer OR email):ti | 35,490 |
| **Databases** | **Step** | **Searches** | **Results** |
|  | #3 | (physical activity OR physical activities OR physical training OR exercise OR exercises OR exercising OR sport OR sports OR step OR steps OR bicycle OR bicycling OR bike OR biking OR walk OR walking OR run OR running OR jog OR jogging OR swim OR swimming OR dance OR dancing OR plyometrics OR resistance training):ti | 82,786 |
|  | #4 | (RCT OR randomized clinical trial OR randomized controlled trial OR randomized trial):ti | 318,964 |
|  | #5 | #1 AND #2 AND #3 AND #4 | 75 |
|  | #6 | (cancer OR cancers OR tumor OR tumors OR neoplasia OR neoplasias OR malignancy OR malignancies OR neoplasm OR neoplasms OR carcinoma OR carcinomas):ab | 201,943 |
|  | #7 | (web OR internet OR text messaging OR digital health OR eHealth OR e-health OR smartphone OR phone OR mobile device OR technology OR mobile health OR mHealth OR m-health OR online OR website OR app OR telephone counseling OR e-counseling OR computer OR email):ab | 143,406 |
|  | #8 | (physical activity OR physical activities OR physical training OR exercise OR exercises OR exercising OR sport OR sports OR step OR steps OR bicycle OR bicycling OR bike OR biking OR walk OR walking OR run OR running OR jog OR jogging OR swim OR swimming OR dance OR dancing OR plyometrics OR resistance training):ab | 220,682 |
|  | #9 | (RCT OR randomized clinical trial OR randomized controlled trial OR randomized trial):ab | 537,195 |
|  | #10 | #6 AND #7 AND #8 AND #9 | 1791 |
|  | #11 | #5 OR #10 | 1817 |

**Databases:** PubMed,Web of Science, Cochrane Library

**Table S2.** Excluded studies with reasons for exclusion

| Study ID and reference | Reason for exclusion | Country |
| --- | --- | --- |
| Keadle et al_ 2021  Keadle S K, Meuter L, Phelan S, et al. Charity-based incentives motivate young adult cancer survivors to increase physical activity: a pilot randomized clinical trial[J]. J Behav Med, 2021,44(5):682-693. | The control group receive a Fitbit-based physical activity intervention with theory-based newsletters. | USA |
| Pope et al_ 2018  Pope Z C, Zeng N, Zhang R, et al. Effectiveness of Combined Smartwatch and Social Media Intervention on Breast Cancer Survivor Health Outcomes: A 10-Week Pilot Randomized Trial[J]. J Clin Med, 2018,7(6). | The control group received electronic health interventions through Facebook, which involved the provision of information related to physical activity. | USA |
| Valle et al_2021  Valle C G, Pinto B M, LaRose J G, et al. Promoting physical activity in young adult cancer survivors using mHealth and adaptive tailored feedback strategies: Design of the Improving Physical Activity after Cancer Treatment (IMPACT) randomized controlled trial[J]. Contemp Clin Trials, 2021,103:106293. | Self-help control group is provided with a tracker, app, scale, video chat, and Facebook to enhance physical activity among cancer survivors. | USA |
| Valle et al_2023  Valle C G, Diamond M A, Heiling H M, et al. Effect of an mHealth intervention on physical activity outcomes among young adult cancer survivors: The IMPACT randomized controlled trial[J]. Cancer, 2023,129(3):461-472. | The control group receives digital tools for self-help to enhance physical activity among cancer survivors. | USA |
| Chapman et al_2018  Chapman J, Fletcher C, Flight I, et al. Pilot randomized trial of a volitional help sheet-based tool to increase leisure time physical activity in breast cancer survivors[J]. Br J Health Psychol, 2018,23(3):723-740. | The control group receives an online volitional help sheet to improve physical activity among breast cancer survivors. | Australia |
| Pinto et al_2021  Pinto B M, Kindred M, Franco R, et al. A 'novel' multi-component approach to promote physical activity among older cancer survivors: a pilot randomized controlled trial[J]. Acta Oncol, 2021,60(8):968-975. | The control group use Fitbit primarily as an intervention component to set weekly step goals. | USA |
| Gell et al_2020  Gell N M, Grover K W, Savard L, et al. Outcomes of a text message, Fitbit, and coaching intervention on physical activity maintenance among cancer survivors: a randomized control pilot trial[J]. J Cancer Surviv, 2020,14(1):80-88. | Control group participants are provided with a Fitbit One device synced to a pre-set account to help maintain physical activity among cancer survivors. | USA |
| Ferrante et al_2022  Ferrante J M, Lulla A, Williamson J D, et al. Patterns of Fitbit Use and Activity Levels Among African American Breast Cancer Survivors During an eHealth Weight Loss Randomized Controlled Trial[J]. Am J Health Promot, 2022,36(1):94-105. | Control group participants wear Fitbit devices, which is a potential intervention for increasing physical activity and improving health outcomes as wearable physical activity accelerometers. | USA |
| Valle et al_2013  Valle C G, Tate D F, Mayer D K, et al. A randomized trial of a Facebook-based physical activity intervention for young adult cancer survivors[J]. J Cancer Surviv, 2013,7(3):355-368. | The control group receives the Facebook-based self-help intervention to enhance physical activity. | USA |
| Finlay et al_2020  Finlay A, Evans H, Vincent A, et al. Optimising Web-Based Computer-Tailored Physical Activity Interventions for Prostate Cancer Survivors: A Randomised Controlled Trial Examining the Impact of Website Architecture on User Engagement[J]. Int J Environ Res Public Health, 2020,17(21). | The control group accessed a static website with oncology guidelines and links to high-quality Australian prostate cancer websites to enhance physical activity. | Australia |
| Chow et al_2021  Chow E J, Doody D R, Di C, et al. Feasibility of a behavioral intervention using mobile health applications to reduce cardiovascular risk factors in cancer survivors: a pilot randomized controlled trial[J]. J Cancer Surviv, 2021,15(4):554-563. | Control participants have self-access to a Fitbit tracker and the Healthwatch360 app to enhance physical activity. | USA |
| Valle et al_2015  Valle C G, Tate D F, Mayer D K, et al. Exploring Mediators of Physical Activity in Young Adult Cancer Survivors: Evidence from a Randomized Trial of a Facebook-Based Physical Activity Intervention[J]. J Adolesc Young Adult Oncol, 2015,4(1):26-33. | The control group receives the Facebook-based self-help intervention to enhance physical activity. | USA |
| Valle et al_2022  Valle C G, Diamond M, Pinto B M, et al. IMPACT: A Randomized Controlled Trial of an mHealth Physical Activity Intervention for Young Adult Cancer Survivors[J]. 2022,31(7):1509. | Control participants have access to self-directed digital tools including an activity tracker, smart scale, and a Facebook group specific to their arm. | USA |
| Ester et al_2023  Ester M, Wagoner CW, Dreger J, et al. Effectiveness of a Self-Monitoring App in Supporting Physical Activity Maintenance Among Rural Canadians With Cancer After an Exercise Oncology Program: Cluster Randomized Controlled Trial. JMIR Cancer. 2023;9:e47187. | The control group accessed an online 12-week exercise intervention to enhance physical activity. | Canada |
| Ariza-Garcia et al_2019  Ariza-Garcia A, Lozano-Lozano M, Galiano-Castillo N, et al. A Web-Based Exercise System (e-CuidateChemo) to Counter the Side Effects of Chemotherapy in Patients With Breast Cancer: Randomized Controlled Trial[J]. J Med Internet Res, 2019,21(7):e14418. | The 6-minute walk test (6MWT) is a valuable assessment for measuring functional capacity, rather than being an outcome measure for PA. | Spain |
| Pinto et al_2022  Pinto B M, Dunsiger S I, Kindred M M, et al. Physical Activity Adoption and Maintenance Among Breast Cancer Survivors: A Randomized Trial of Peer Mentoring[J]. Ann Behav Med, 2022,56(8):842-855. | The level of moderate-to-vigorous physical activity is presented in a graphical format that could not be extracted, and no response is received from the corresponding author upon communication. | USA |
| Hartman et al_2018  Hartman S J, Nelson S H, Weiner L S. Patterns of Fitbit Use and Activity Levels Throughout a Physical Activity Intervention: Exploratory Analysis from a Randomized Controlled Trial[J]. JMIR Mhealth Uhealth, 2018,6(2):e29. | The level of moderate-to-vigorous physical activity is presented in a graphical format that could not be extracted, and no response is received from the corresponding author upon communication. | USA |
| Pinto et al_2013  Pinto B M, Dunsiger S, Waldemore M. Physical activity and psychosocial benefits among breast cancer patients[J]. Psychooncology, 2013,22(10):2193-2199. | The level of moderate-to-vigorous physical activity are presented in a graphical format that could not be extracted, and no response is received from the corresponding author upon communication. | USA |
| Lee et al_2014  Lee M K, Yun Y H, Park H A, et al. A Web-based self-management exercise and diet intervention for breast cancer survivors: pilot randomized controlled trial[J]. Int J Nurs Stud, 2014,51(12):1557-1567. | The outcome measure for physical activity levels is presented as the percentage of participants achieving moderate-intensity aerobic exercise for at least 150 minutes per week (n %).After communicating the issue with the corresponding author, no feedback is received. | Korea |
| Yun et al_2020  Yun Y H, Lim C I, Lee E S, et al. Efficacy of health coaching and a web-based program on physical activity, weight, and distress management among cancer survivors: A multi-centered randomised controlled trial[J]. 2020,29(7):1105-1114. | The outcome measure for physical activity levels is presented using a percentage format, and no feedback is received from the corresponding author after communication. | Korea |
| Rees-Punia et al_2022  Rees-Punia E, Leach C R, Westmaas J L, et al. Pilot Randomized Controlled Trial of Feasibility, Acceptability, and Preliminary Efficacy of a Web-Based Physical Activity and Sedentary Time Intervention for Survivors of Physical Inactivity-Related Cancers[J]. Int J Behav Med, 2022,29(2):220-229. | The level of moderate-to-vigorous physical activity (MVPA) and light physical activity (LPA) are presented in a graphical format that could not be extracted, and no response is received from the corresponding author upon communication. | USA |
| Blair et al_2021  Blair C K, Harding E, Wiggins C, et al. A Home-Based Mobile Health Intervention to Replace Sedentary Time With Light Physical Activity in Older Cancer Survivors: Randomized Controlled Pilot Trial[J]. JMIR Cancer, 2021,7(2):e18819. | Steps per 15 hours awake, light-intensity physical activity, and moderate-intensity physical activity are expressed as least square means, and no feedback is received from the corresponding author after communication. | USA |
| Terranova et al_2022  Terranova C O, Winkler E, Healy G N, et al. Dietary and Physical Activity Changes and Adherence to WCRF/AICR Cancer Prevention Recommendations following a Remotely Delivered Weight Loss Intervention for Female Breast Cancer Survivors: The Living Well after Breast Cancer Randomized Controlled Trial[J]. J Acad Nutr Diet, 2022,122(9):1644-1664. | Activity counts, moderate-to-vigorous physical activity and walking/running are expressed as adjusted means, and no feedback was received from the corresponding author after communication. | Australia |
| Gomersall et al_2019  Gomersall S R, Skinner T L, Winkler E, et al. Feasibility, acceptability and efficacy of a text message-enhanced clinical exercise rehabilitation intervention for increasing 'whole-of-day' activity in people living with and beyond cancer[J]. BMC Public Health, 2019,19(Suppl 2):542. | The outcome measures related to physical activity levels are expressed as adjusted means, and no feedback is received from the corresponding author after communication. | Australia |
| Mayer et al_2018  Mayer D K, Landucci G, Awoyinka L, et al. SurvivorCHESS to increase physical activity in colon cancer survivors: can we get them moving?[J]. J Cancer Surviv, 2018,12(1):82-94. | The outcome measures related to physical activity levels are presented in a graphical format that could not be extracted, and no response is received from the corresponding author upon communication. | USA |
| Ormel et al_2018  Ormel H L, van der Schoot G, Westerink N L, et al. Self-monitoring physical activity with a smartphone application in cancer patients: a randomized feasibility study (SMART-trial)[J]. Support Care Cancer, 2018,26(11):3915-3923. | Total minutes of physical activity are expressed as median and interquartile range (IQR), and no feedback is received from the corresponding author after communication. | Netherlands |
| Ochi et al_2022  Ochi E, Tsuji K, Narisawa T, et al. Cardiorespiratory fitness in breast cancer survivors: a randomised controlled trial of home-based smartphone supported high intensity interval training[J]. BMJ Support Palliat Care, 2022,12(1):33-37. | The outcome measures related to physical activity are only secondary endpoints in this study. | Japan |
| Waller et al_2022  Waller E, Sutton P, Rahman S, et al. Prehabilitation with wearables versus standard of care before major abdominal cancer surgery: a randomised controlled pilot study (trial registration: NCT04047524)[J]. Surg Endosc, 2022,36(2):1008-1017. | The 6-minute walk test (6MWT) is a valuable assessment for measuring functional capacity, rather than being an outcome measure for PA. | USA |
| Short et al_2014  Short C E, Coysh C, Vandelanotte C, et al. PROMOTING PHYSICAL ACTIVITY AMONG BREAST CANCER SURVIVORS USING COMPUTER-TAILORED ONLINE INTERVENTIONS: IMOVE MORE FOR LIFE RCT[J]. 2014,10:261. | It is a conference abstract. | Australia |
| Ochi et al_2022  Ochi E, Tsuji K, Narisawa T, et al. Effect of home-based smartphone-supported high-intensity interval training on cardiorespiratory fitness in breast cancer survivors: a randomized controlled trial of the habit-B program[J]. 2022,82(4 SUPPL). | It is a conference abstract. | USA |
| Kenfield et al_2018  Kenfield S A, Blarigan E V, Ameli N, et al. Prostate 8 study: a pilot randomized controlled trial (RCT) of a web-based lifestyle intervention versus control group among men with prostate cancer[J]. 2018,36(6). | It is a conference abstract. | USA |
| Ma et al_2022  Ma D, Au K, Liu Z, et al. Randomised trial on the impact of virtual-based multimodal training programme on physical fitness and quality of life in patients late post-haematopoietic stem cell transplant (HCT)[J]. 2022,57:80. | It is a conference abstract. | Netherlands |
| Arrieta et al_2018  Arrieta H, Astrugue C, Regueme S, et al. Randomized clinical trial of telephoned-based physical activity intervention in onco-geriatric patients[J]. 2018,36(15). | It is a conference abstract. | USA |
| Murphy et al_2023  Murphy A C, Farouque O, Koshy A N, et al. Randomized Controlled Trial of a Smartphone-Based Intervention to Enhance 6-Minute Walk Distance During Breast Cancer Treatment: The SMART-BREAST Trial[J]. 2023,147(7):614-616. | It is a conference abstract. | Australia |
| Schwartz et al_2016  Schwartz L A, Daniel L C, Butler E, et al. A pilot text messaging intervention for adolescents and young adults recently off treatment for cancer  [J]. 2016,58(2):S30. | It is a conference abstract. | USA |
| Salchow et al_2017  Salchow J, Jensen W, Koch B, et al. Effects of a structured intervention program to improve physical activity of adolescents and young adult cancer survivors (AYAs)-Interim analysis of a randomized controlled trial-Motivate AYA-MAYA trial[J]. 2017,40:215. | It is a conference abstract. | Germany |
| Maxwell-Smith et al_2018  Maxwell-Smith C M, Cohen P A, Platell C, et al. Increasing physical activity in cancer survivors at cardiovascular risk using Fitbits: randomized controlled trial[J]. 2018,36(7). | It is a conference abstract. | USA |
| Zamorano et al_2021  Zamorano A , Wilson E , Liu J , et al. Low quality of life and activity levels persist in obese endometrial cancer survivors despite participation in a behavioral weight loss intervention[J]. Gynecologic Oncology, 2021, 162:S201. | It is a conference abstract. | USA |
| Kim et al_2023  Kim SW, Lim JY, Yeo SM, et al. EFFECTIVENESS of A DIGITAL THERAPEUTICS-BASED PERSONALIZED REHABILITATION in PATIENTS with COLORECTAL CANCER after SURGERY for 1 YEAR. Supportive care in cancer. 2023;31:S36‐S37. | It is a conference abstract. | Korea |
| Chung et al_2020  Chung I Y, Jung M, Park Y R, et al. Exercise Promotion and Distress Reduction Using a Mobile App-Based Community in Breast Cancer Survivors[J]. Front Oncol, 2019,9:1505. | The study is non-randomized and prospective. | Korea |
| Spees et al_2019  Spees C K, Braun A C, Hill E B, et al. Impact of a Tailored Nutrition and Lifestyle Intervention for Overweight Cancer Survivors on Dietary Patterns, Physical Activity, Quality of Life, and Cardiometabolic Profiles[J]. J Oncol, 2019,2019:1503195. | This is a pre-post interventional study that is non-randomized. | USA |
| Frensham et al_ 2020  Frensham L J, Parfitt G, Dollman J. Predicting Engagement With Online Walking Promotion Among Metropolitan and Rural Cancer Survivors[J]. Cancer Nurs, 2020,43(1):52-59. | It is a quasi-randomized controlled intervention trial. | Australia |
| Trinh et al_ 2018  Trinh L, Arbour-Nicitopoulos K P, Sabiston C M, et al. RiseTx: testing the feasibility of a web application for reducing sedentary behavior among prostate cancer survivors receiving androgen deprivation therapy[J]. Int J Behav Nutr Phys Act, 2018,15(1):49. | The study is a prospective and single-arm design. | Canada |
| Schrier et al_2021  Schrier E, Xiong N, Thompson E, et al. Stepping into survivorship pilot study: Harnessing mobile health and principles of behavioral economics to increase physical activity in ovarian cancer survivors[J]. Gynecol Oncol, 2021,161(2):581-586. | It is a single-arm pilot study without a control group. | USA |
| Spark et al_2015  Spark L C, Fjeldsoe B S, Eakin E G, et al. Efficacy of a Text Message-Delivered Extended Contact Intervention on Maintenance of Weight Loss, Physical Activity, and Dietary Behavior Change[J]. JMIR Mhealth Uhealth, 2015,3(3):e88. | It is a single-group, pre-post designed study. | Australia |
| Salchow et al_2021  Salchow J, Koch B, Mann J, et al. Effects of a structured counselling-based intervention to improve physical activity behaviour of adolescents and young adult cancer survivors - the randomized phase II Motivate AYA - MAYA trial[J]. Clin Rehabil, 2021,35(8):1164-1174. | Adolescent and young adult cancer survivors aged 15 to 39 years are the population, not adults. | Germany |
| Kanera et al_2016  Kanera I M, Bolman C A, Willems R A, et al. Lifestyle-related effects of the web-based Kanker Nazorg Wijzer (Cancer Aftercare Guide) intervention for cancer survivors: a randomized controlled trial[J]. J Cancer Surviv, 2016,10(5):883-897. | The study population in this article is consistent with that of "Long-term effects of a web-based cancer aftercare intervention on moderate physical activity and vegetable consumption among early cancer survivors: a randomized controlled trial." | Netherlands |
| Chan et al_2023  Chan D N S, Chow K M, Anderson D J, et al. Cultural Adaptation of the Younger Women's Wellness After Cancer Program for Younger Chinese Women With Breast Cancer: A Pilot Randomized Controlled Trial[J]. 2023. | The assessment of physical activity levels in this study is based on questionnaire scores as the outcome measure. | Netherlands |
| Walsh et al_2021  Walsh J C, Richmond J, Mc Sharry J, et al. Examining the Impact of an mHealth Behavior Change Intervention With a Brief In-Person Component for Cancer Survivors With Overweight or Obesity: Randomized Controlled Trial[J]. 2021,9(7):e24915. | The assessment of physical activity levels in this study is based on questionnaire scores as the outcome measure. | Ireland |
| Webb et al_2019  Webb J, Fife-Schaw C, Ogden J. A randomised control trial and cost-consequence analysis to examine the effects of a print-based intervention supported by internet tools on the physical activity of UK cancer survivors[J]. Public Health, 2019,171:106-115. | The assessment of physical activity levels in this study is based on questionnaire scores as the outcome measure. | UK |
| Ligibel et al_2012  Ligibel J A, Meyerhardt J, Pierce J P, et al. Impact of a telephone-based physical activity intervention upon exercise behaviors and fitness in cancer survivors enrolled in a cooperative group setting[J]. 2012,132(1):205-213. | The outcome measures of physical activity levels after the intervention are not presented and no response is received from the corresponding author upon communication. | USA |
| Hatchett et al_2013  Hatchett A, Hallam J S, Ford M A. Evaluation of a social cognitive theory-based email intervention designed to influence the physical activity of survivors of breast cancer[J]. Psychooncology, 2013,22(4):829-836. | The measure of physical activity levels in this study using "days per week" for moderate physical activity may not be sensitive enough to detect changes in physical activity levels, and no response is received from the corresponding author upon communication. | USA |
| Golsteijn et al_2023  Golsteijn RHJ, Bolman C, Peels DA, Volders E, de Vries H, Lechner L. Long-term efficacy of a computer-tailored physical activity intervention for prostate and colorectal cancer patients and survivors: A randomized controlled trial [published online ahead of print, 2023 Aug 15]. J Sport Health Sci. 2023;S2095-2546(23)00075-3. | This article presents the follow-up results of the "Short-term efficacy of a computer-tailored physical activity intervention for prostate and colorectal cancer patients and survivors: a randomized controlled trial." | Netherlands |
| Valle et al_2023  Valle CG, Diamond MA, Heiling HM, et al. Physical activity maintenance among young adult cancer survivors in an mHealth intervention: Twelve-month outcomes from the IMPACT randomized controlled trial. Cancer Med. 2023;12(15):16502-16516. | This article presents the follow-up results of the "Effect of an mHealth intervention on physical activity outcomes among young adult cancer survivors: The IMPACT randomized controlled trial" . | USA |
| Jung et al_2023  Jung M, Lee SB, Lee JW, et al. The Impact of a Mobile Support Group on Distress and Physical Activity in Breast Cancer Survivors: Randomized, Parallel-Group, Open-Label, Controlled Trial. J Med Internet Res. 2023;25:e47158. Published 2023 Aug 7. | The outcome measures for physical activity levels after the intervention are not presented as mean (SD) or mean, and no response has been received from the corresponding author despite attempts at communication. | Korea |
| Paxton et al_2017  Paxton R J, Hajek R, Newcomb P, et al. A Lifestyle Intervention via Email in Minority Breast Cancer Survivors: Randomized Parallel-Group Feasibility Study[J]. JMIR Cancer, 2017,3(2):e13. | The outcome measures for physical activity levels after the intervention are only presented as chang (SE), and no response has been received from the corresponding author despite attempts at communication. | USA |
| Neil-Sztramko et al_2019  Neil-Sztramko S E, Belita E, Levinson A J, et al. Evaluation of an online knowledge translation intervention to promote cancer risk reduction behaviours: findings from a randomized controlled trial[J]. BMC Cancer, 2019,19(1):1138. | The outcome measures for physical activity levels after the intervention are only presented as odds ratios, and no response has been received from the corresponding author despite multiple attempts at communication. | Canada |
| Sheean et al_2021  Sheean P, Matthews L, Visotcky A, et al. Every Day Counts: a randomized pilot lifestyle intervention for women with metastatic breast cancer[J]. Breast Cancer Res Treat, 2021,187(3):729-741. | Participants in the control group receive a monthly text message thanking them for their participation in the study and reminding them of their intervention start. | USA |
| Gehring et al_2018  Gehring K, Kloek C J, Aaronson N K, et al. Feasibility of a home-based exercise intervention with remote guidance for patients with stable grade II and III gliomas: a pilot randomized controlled trial[J]. Clin Rehabil, 2018,32(3):352-366. | Patients in control group also receive bi-monthly phone calls from the research assistant during which general questions about their health are asked to encourage physical activity. | Dutch |
| Pinto et al_2013  Pinto B M, Dunsiger S, Waldemore M. Physical activity and psychosocial benefits among breast cancer patients[J]. Psychooncology, 2013,22(10):2193-2199. | The level of moderate-to-vigorous physical activity are presented in a graphical format that could not be extracted, and no response is received from the corresponding author upon communication. | USA |


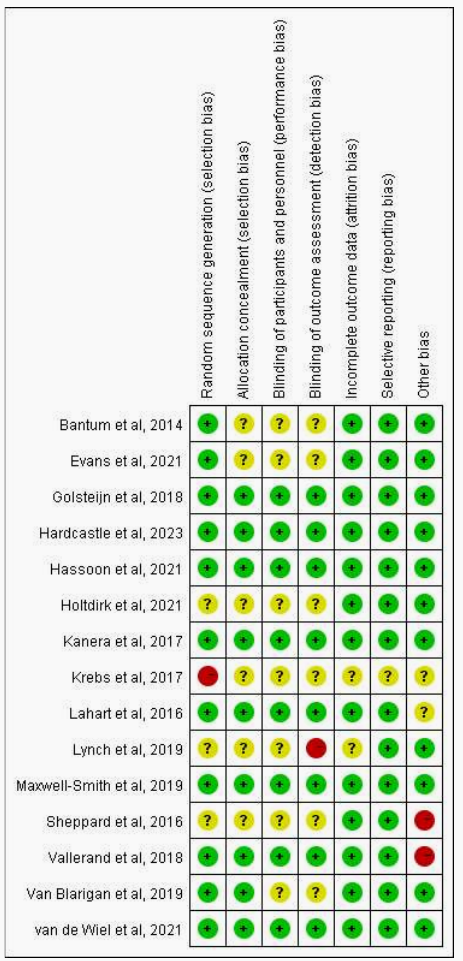


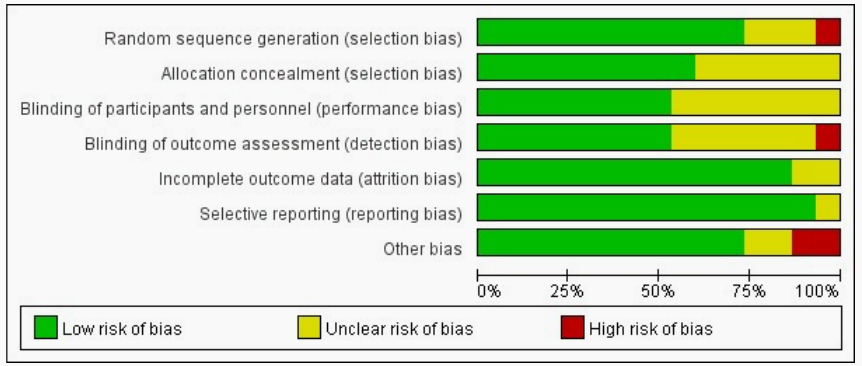


**Figure S1.** Risk of bias summary of the included studies

Table S3. The Cochrane tool for assessing risk of bias


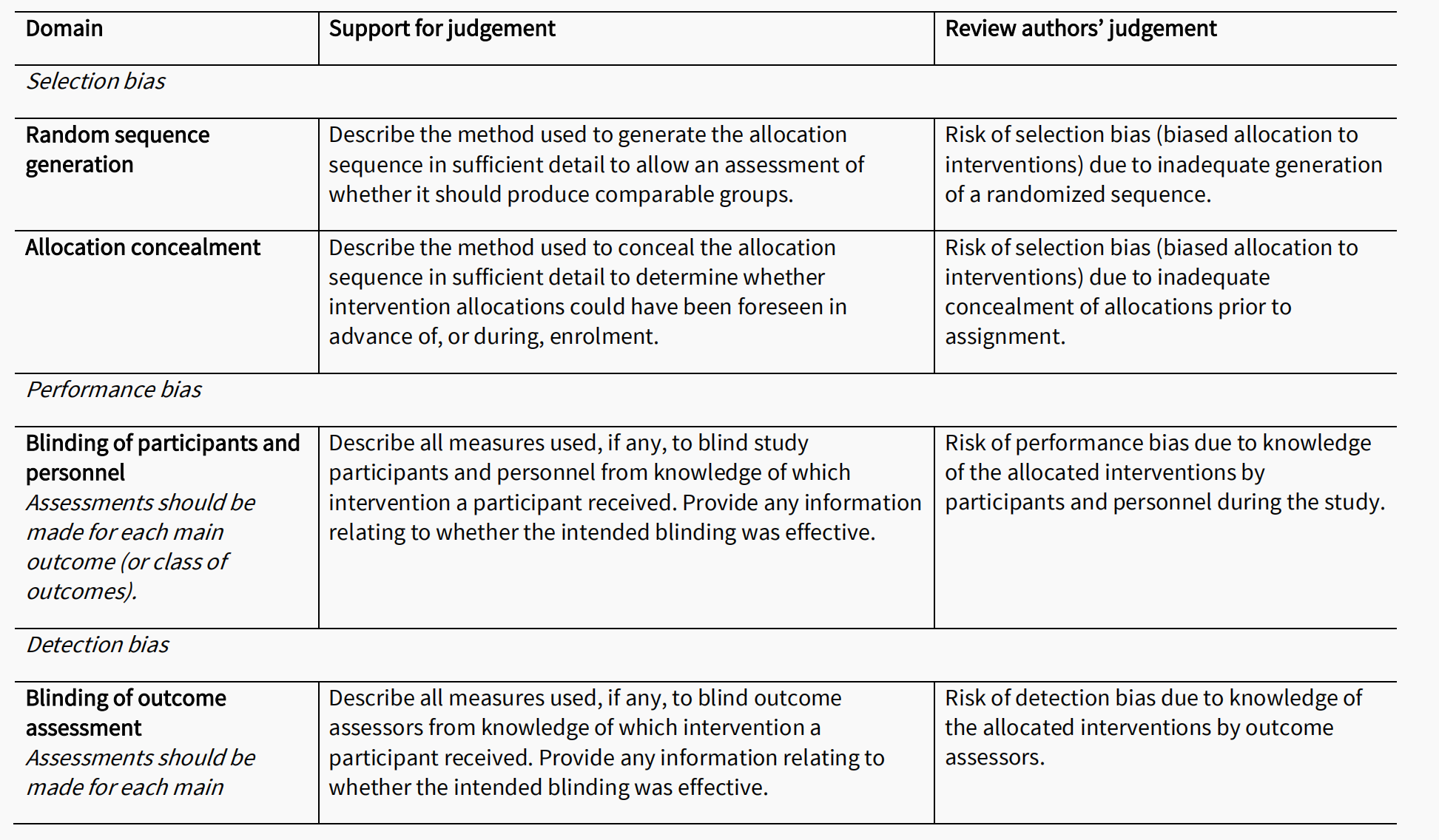


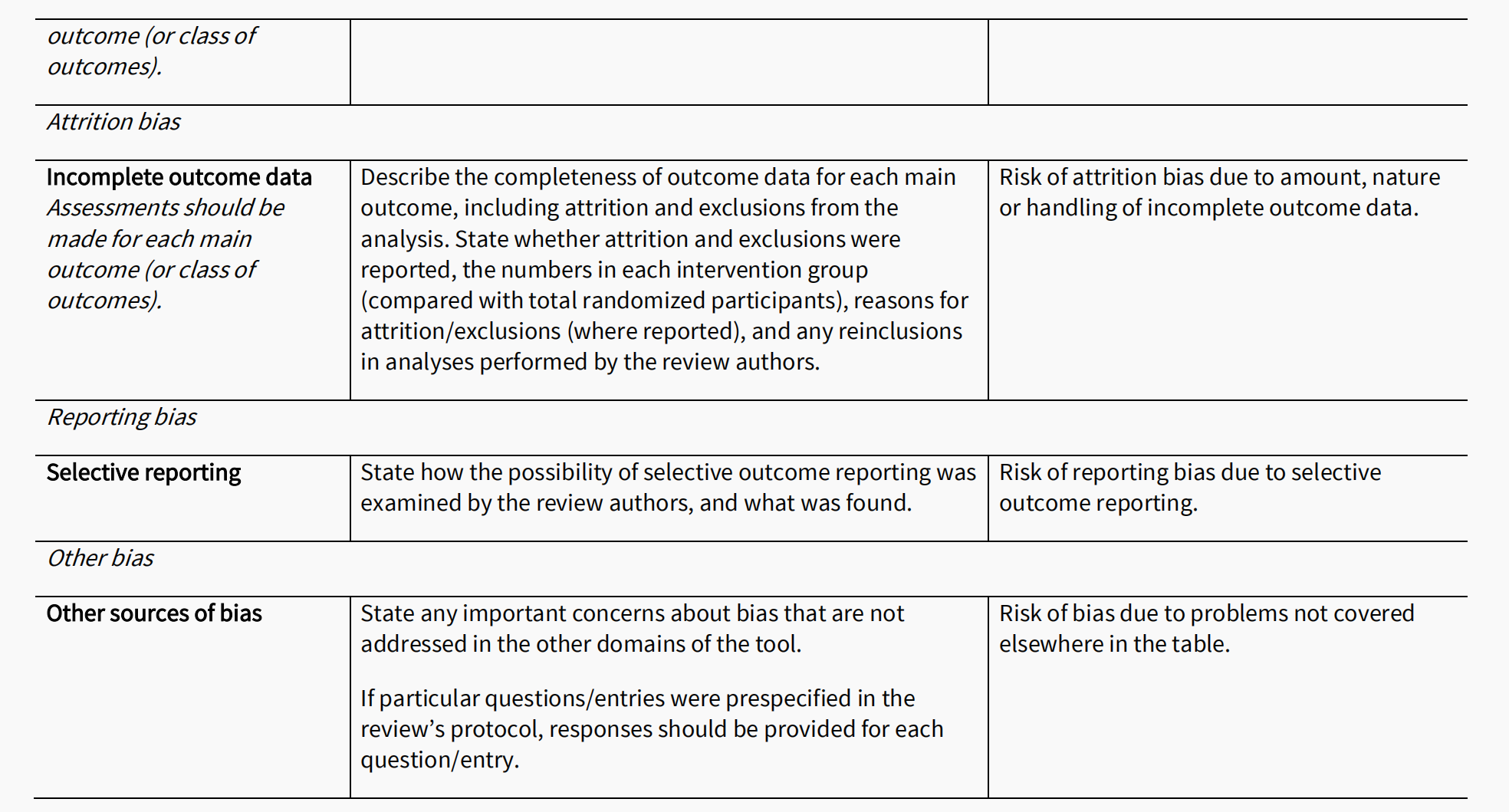


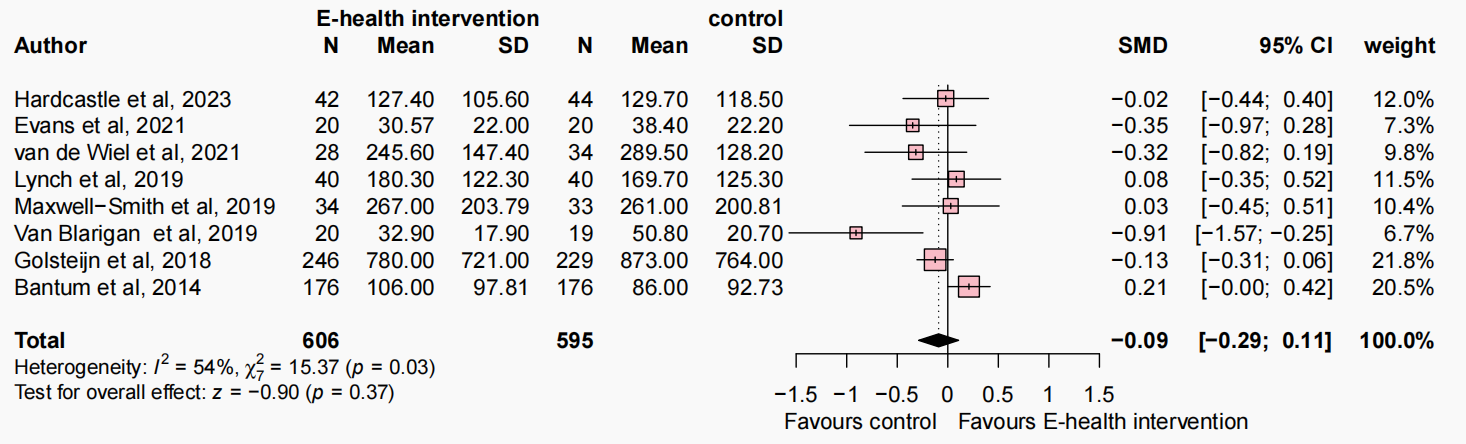


**Figure S2.** Forest plot of baseline characteristics for the effects of E-health interventions on MVPA in cancer survivors (n = 8).


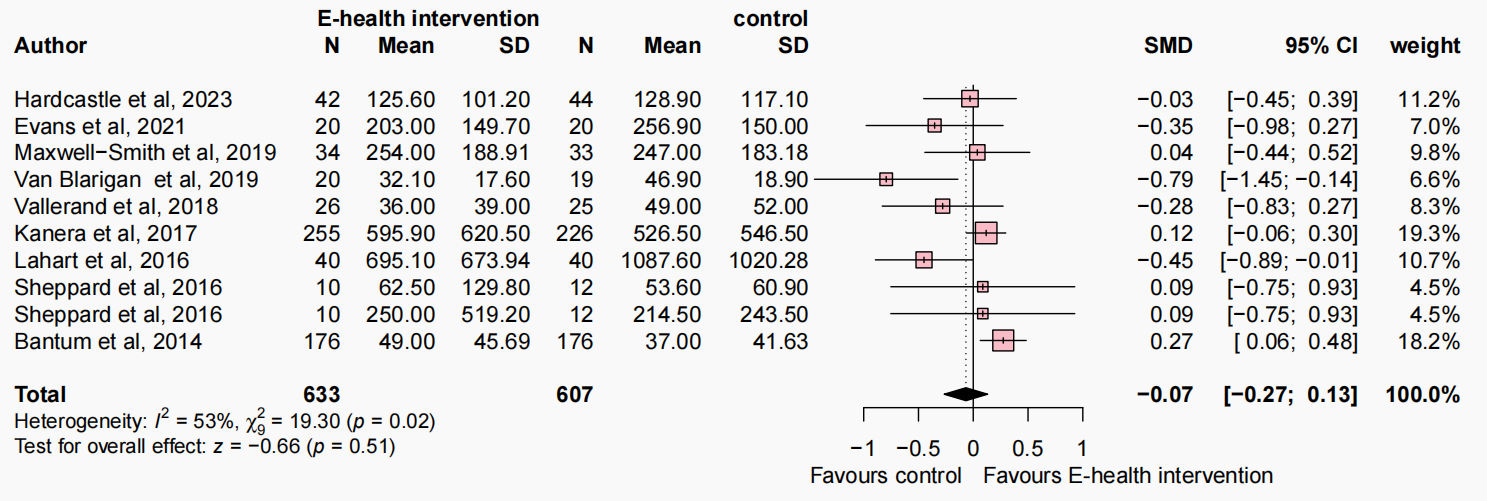


**Figure S3.** Forest plot of baseline characteristics for the effects of E-health interventions on MPA in cancer survivors (n = 9).


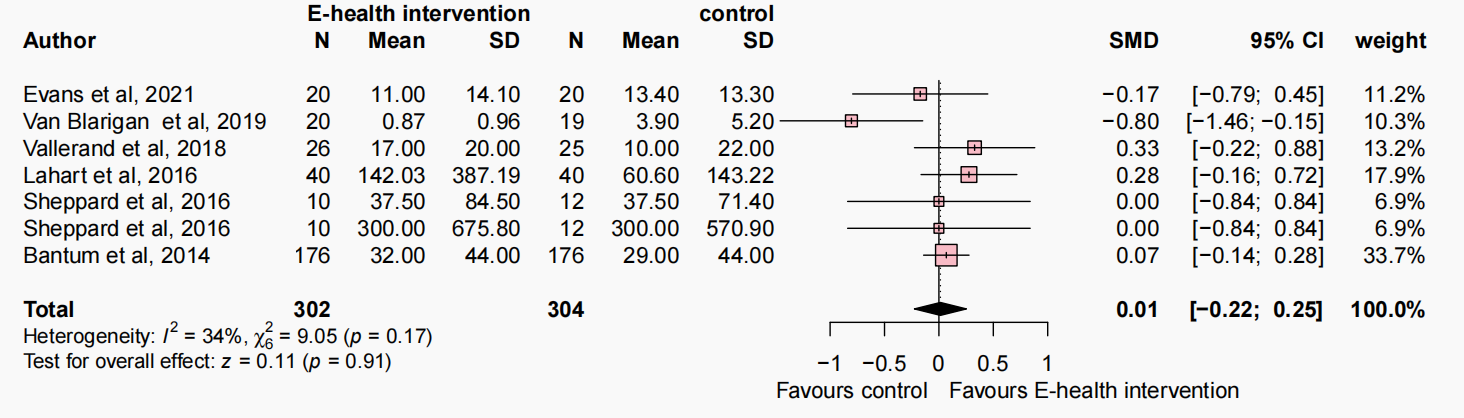


**Figure S4.** Forest plot of baseline characteristics for the effects of E-health interventions on VPA in cancer survivors (n = 6).


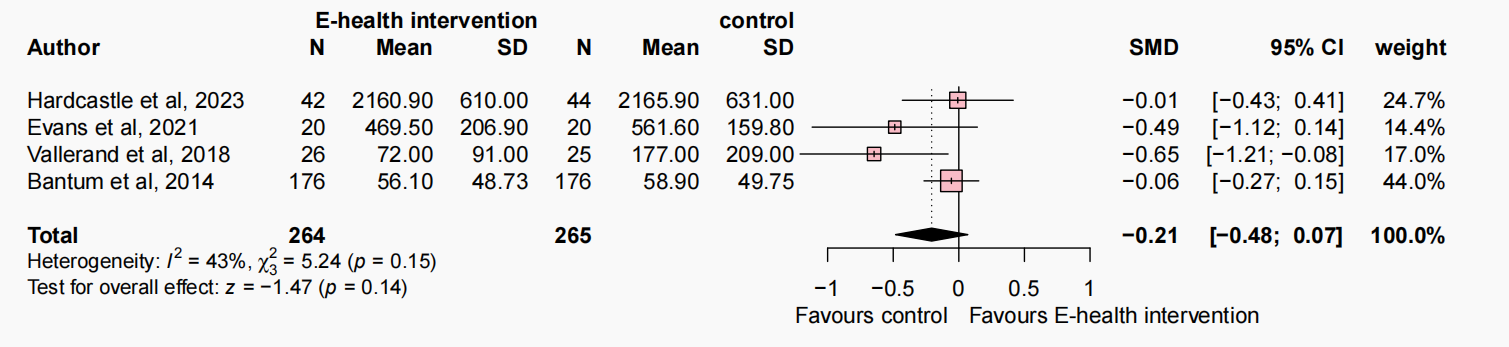


**Figure S5.** Forest plot of baseline characteristics for the effects of E-health interventions on LPA in cancer survivors (n = 4).


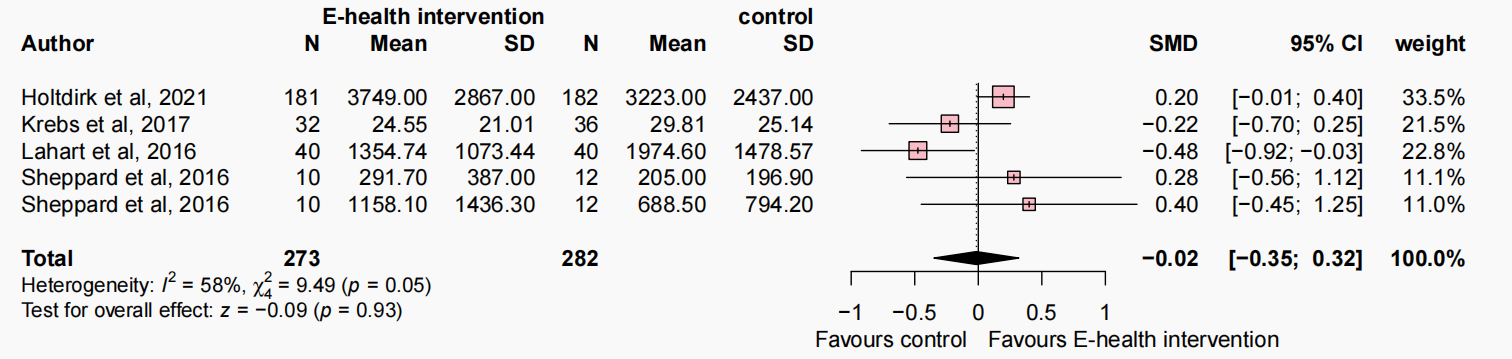


**Figure S6.** Forest plot of baseline characteristics for the effects of E-health interventions on TPA in cancer survivors (n = 4).


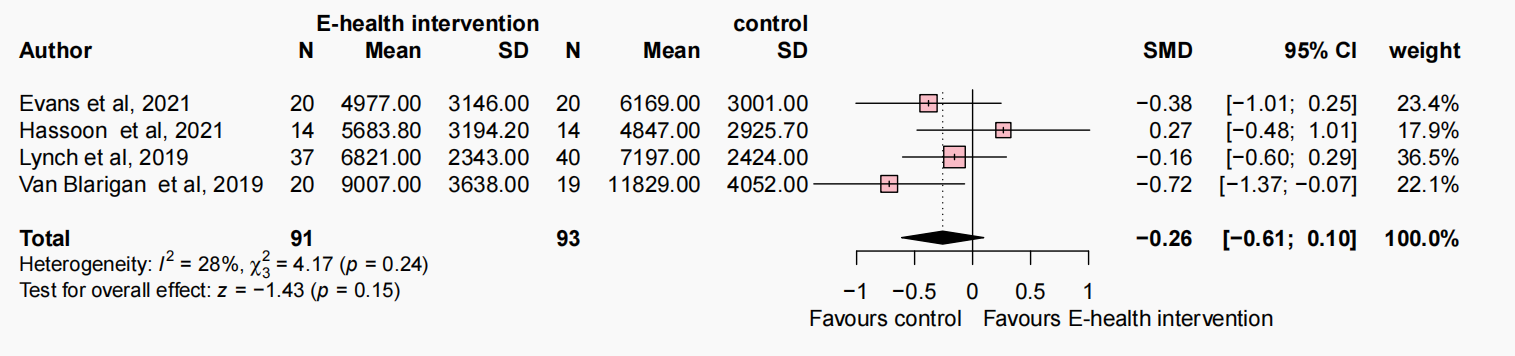


**Figure S7.** Forest plot of baseline characteristics for the effects of E-health interventions on steps in cancer survivors (n = 4).


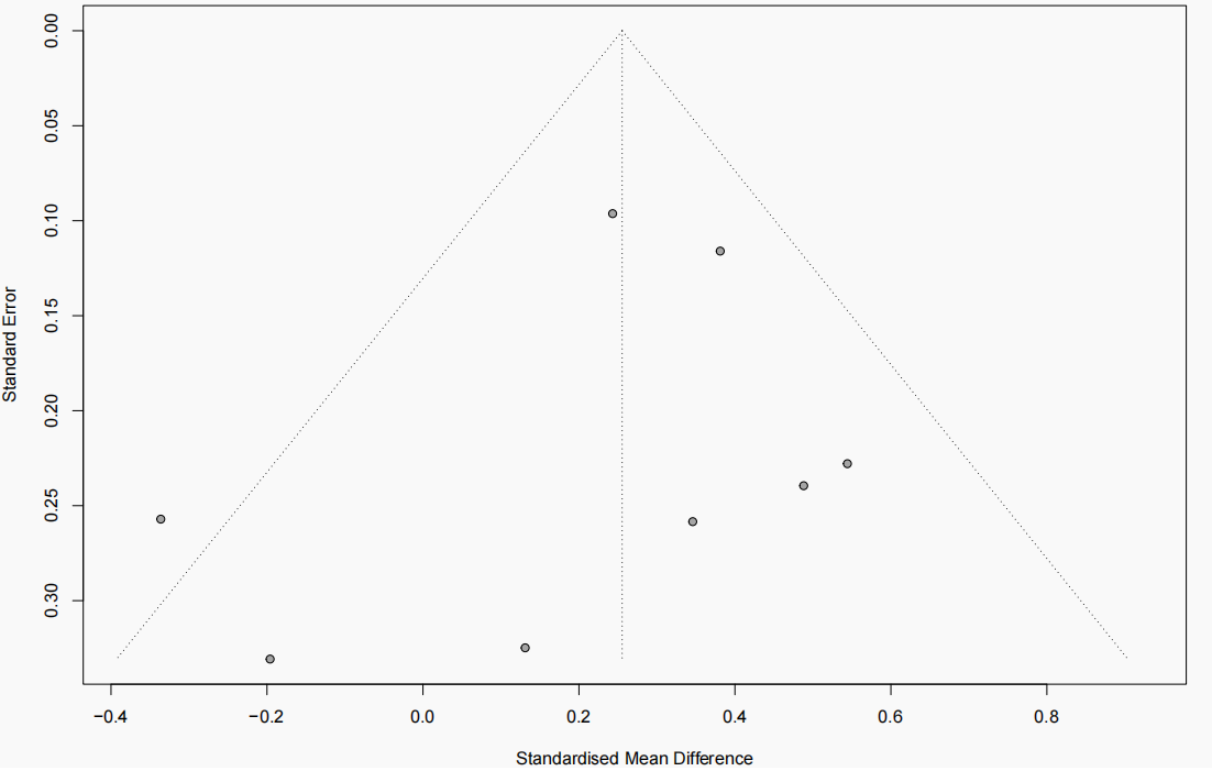


t = -0.76, df = 6, p = 0.4780

**Figure S8.** Funnel plot comparison of E-health intervention for MVPA in cancer survivors (n = 8).


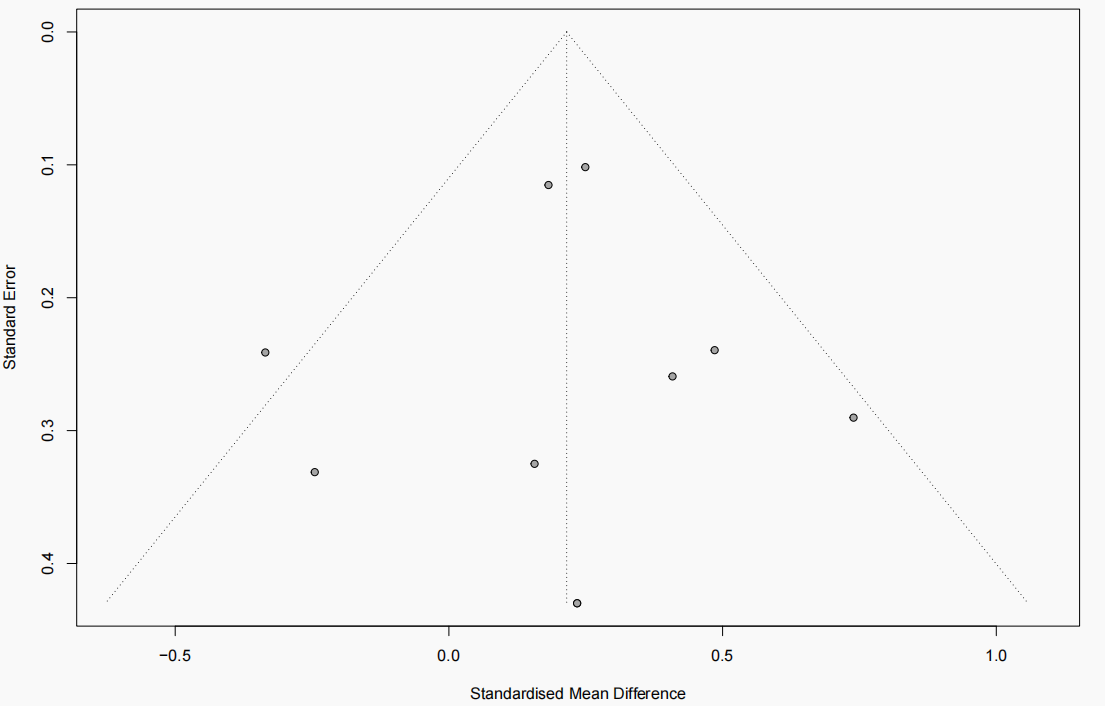


t = -0.07, df = 8, p = 0.9439

**Figure S9.** Funnel plot comparison of E-health intervention for MPA in cancer survivors (n = 9).


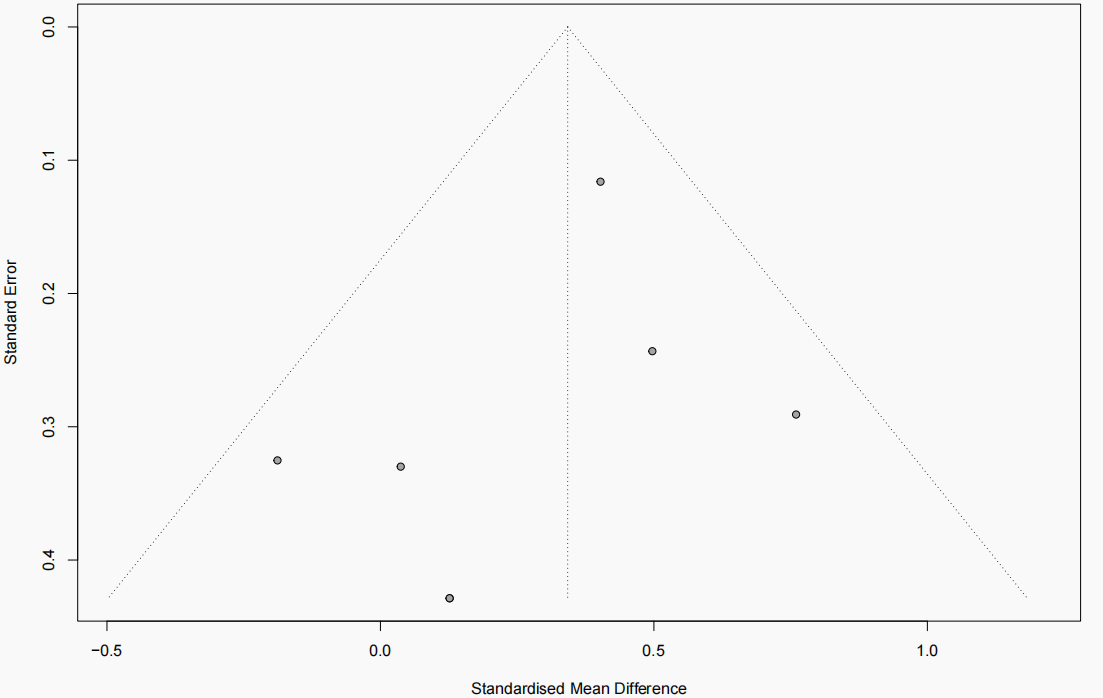


t = -1.04, df = 5, p= 0.3470

**Figure S10.** Funnel plot comparison of E-health intervention for VPA in cancer survivors (n = 6).


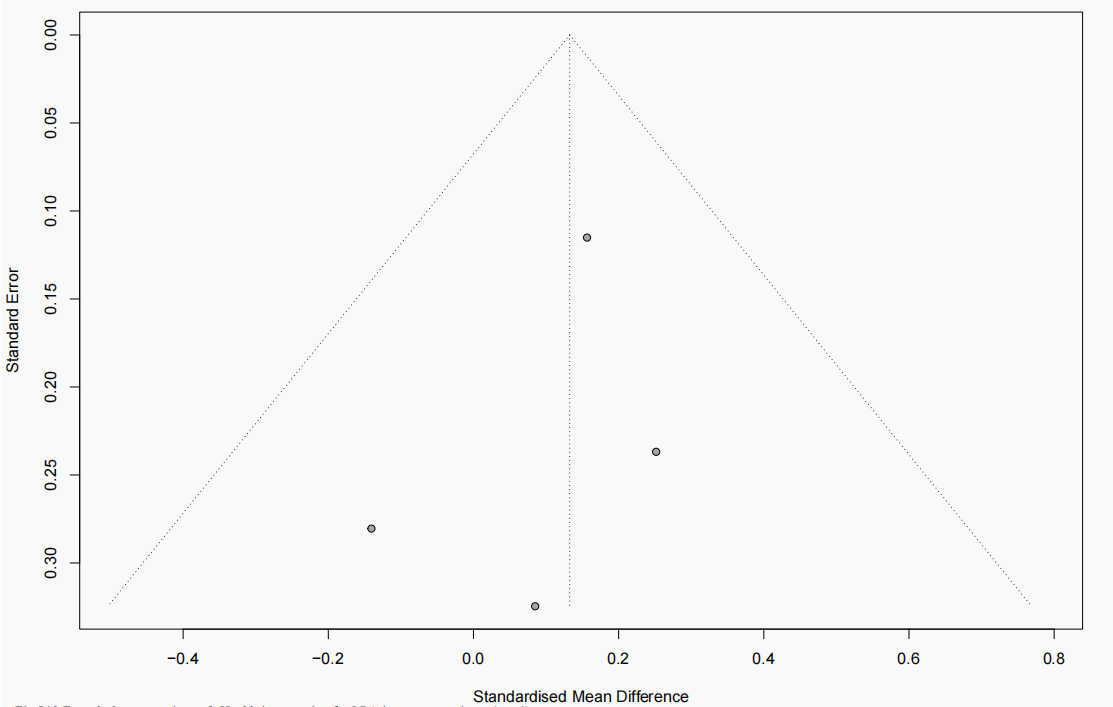
t = -0.69, df = 2, p = 0.5605

**Figure S11.** Funnel plot comparison of E-health intervention for LPA in cancer survivors (n = 4).


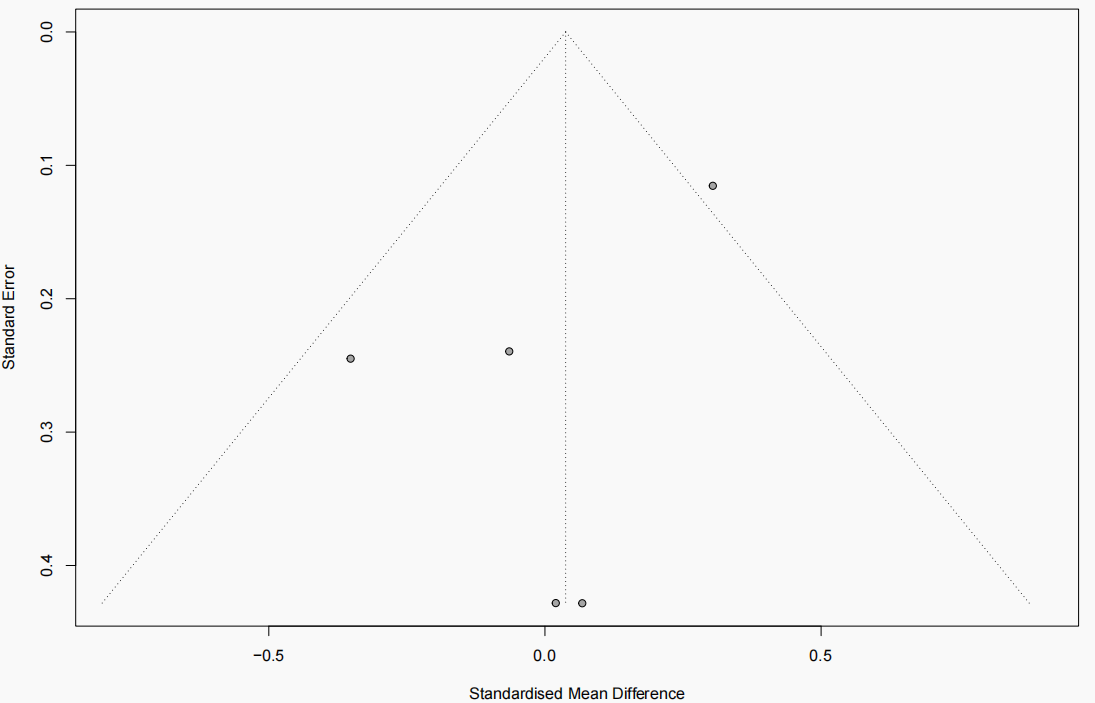


t = -1.48, df = 3, p = 0.2356

**Figure S12.** Funnel plot comparison of E-health intervention for TPA in cancer survivors (n = 4).


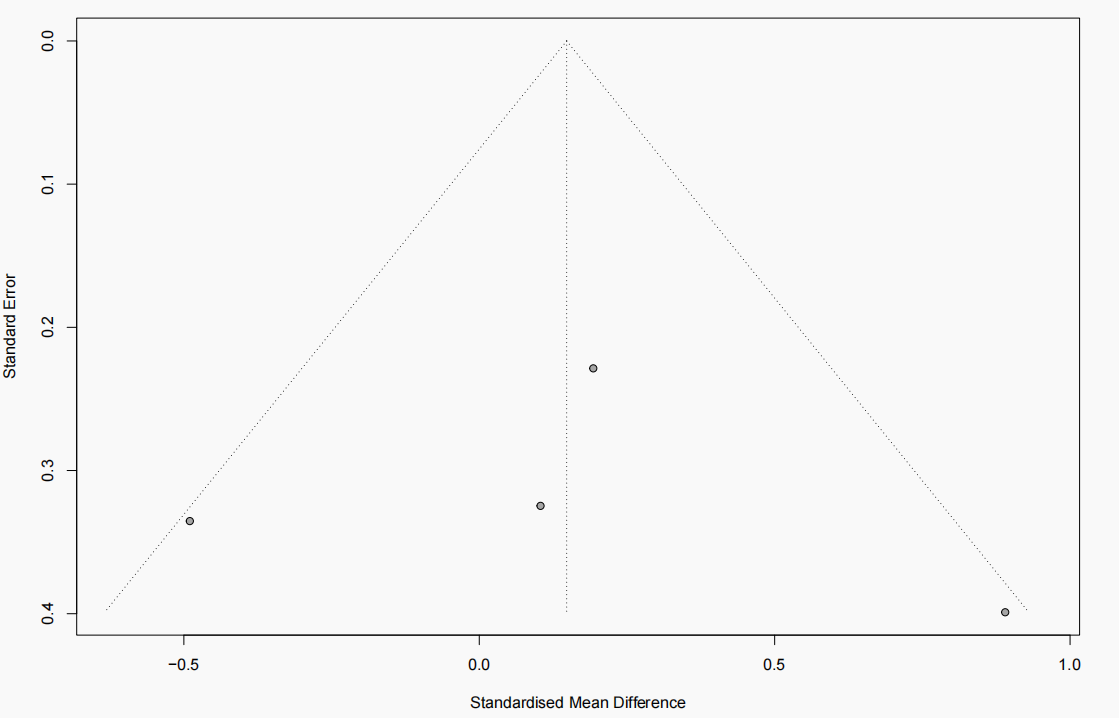


t = 0.24, df = 2, p = 0.8326

**Figure S13.** Funnel plot comparison of E-health intervention for steps in cancer survivors (n =4 ).


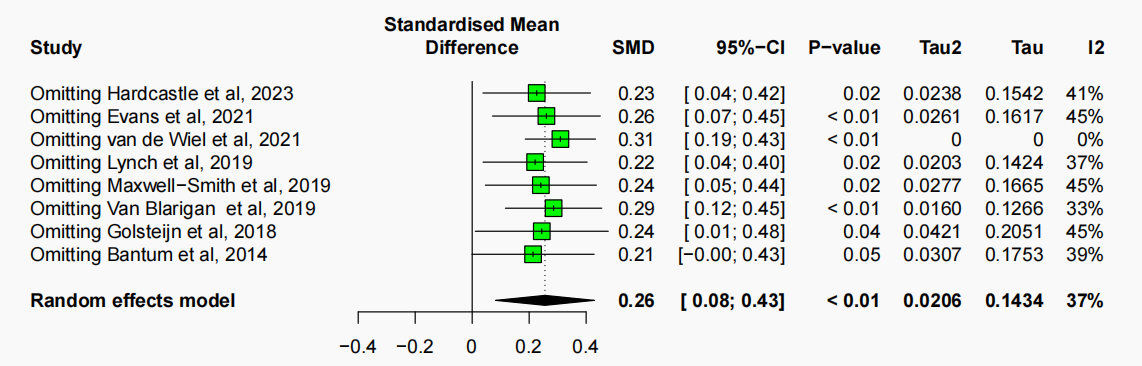


**Figure S14.** Sensitivity analysis on the effect of E-health intervention on MVPA in cancer survivors (n = 8).


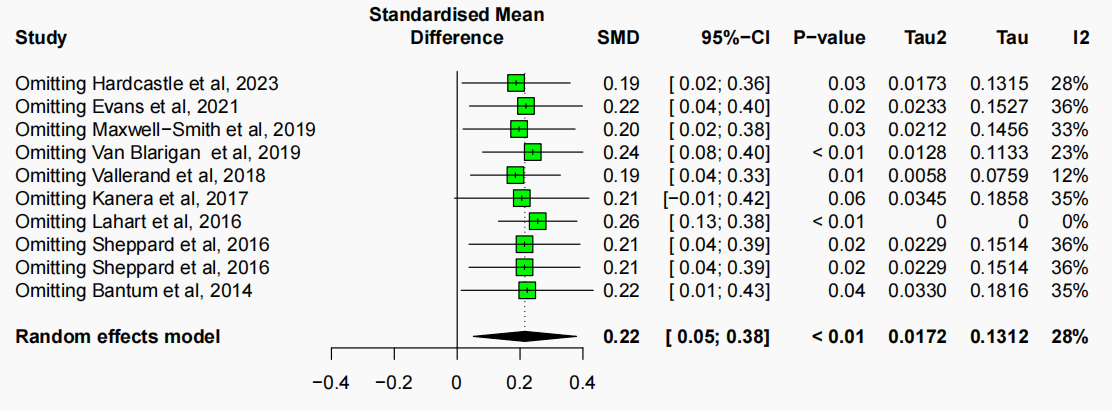


**Figure S15.** Sensitivity analysis on the effect of E-health intervention on MPA in cancer survivors (n = 9).


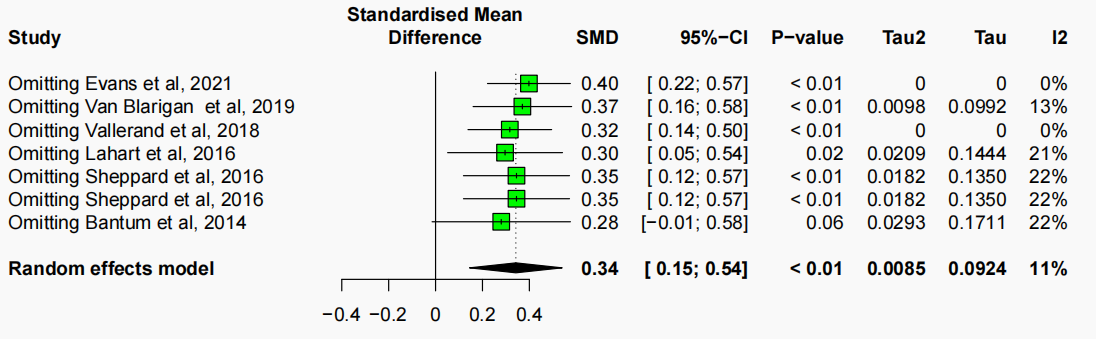


**Figure S16.** Sensitivity analysis on the effect of E-health intervention on VPA in cancer survivors (n = 6).


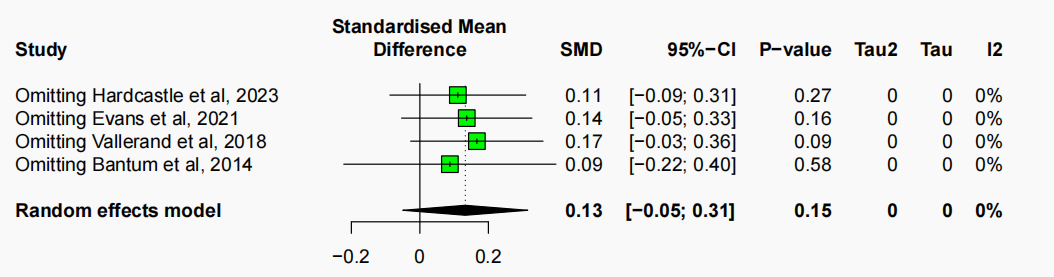


**Figure S17.** Sensitivity analysis on the effect of E-health intervention on LPA in cancer survivors (n = 4).


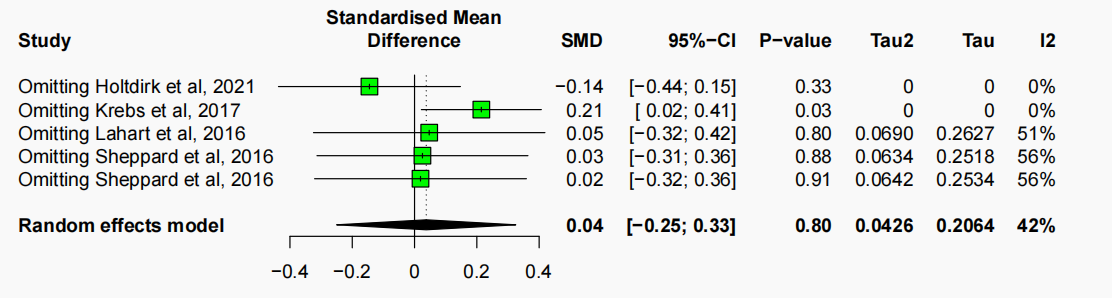


**Figure S18.** Sensitivity analysis on the effect of E-health intervention on TPA in cancer survivors (n = 4).


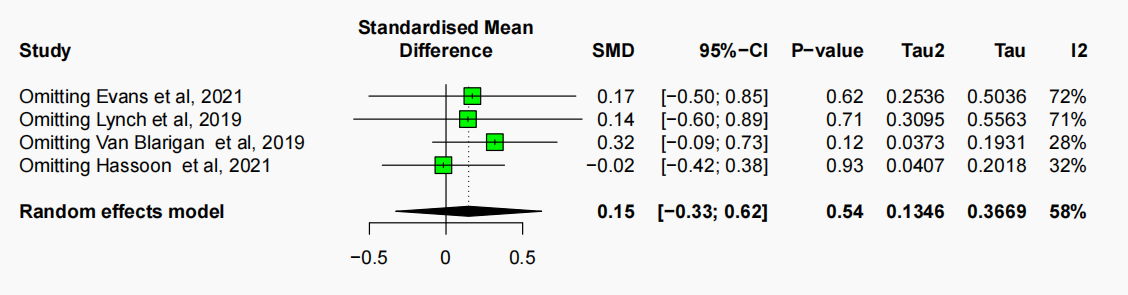


**Figure S19.** Sensitivity analysis on the effect of E-health intervention on steps in cancer survivors (n = 4).


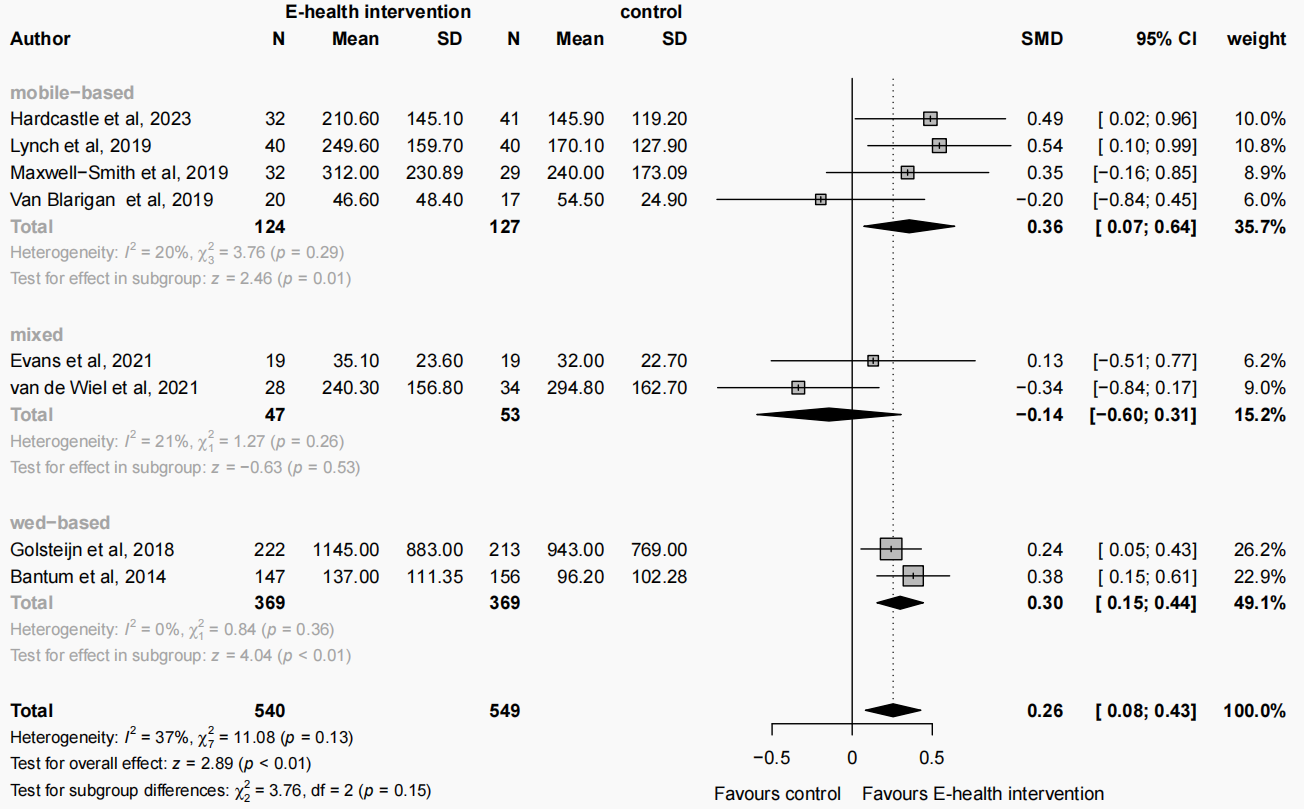


**Figure S20.** Subgroup analysis of E-health intervention method in the effect of E-health interventions on MVPA in cancer survivors(n=8).


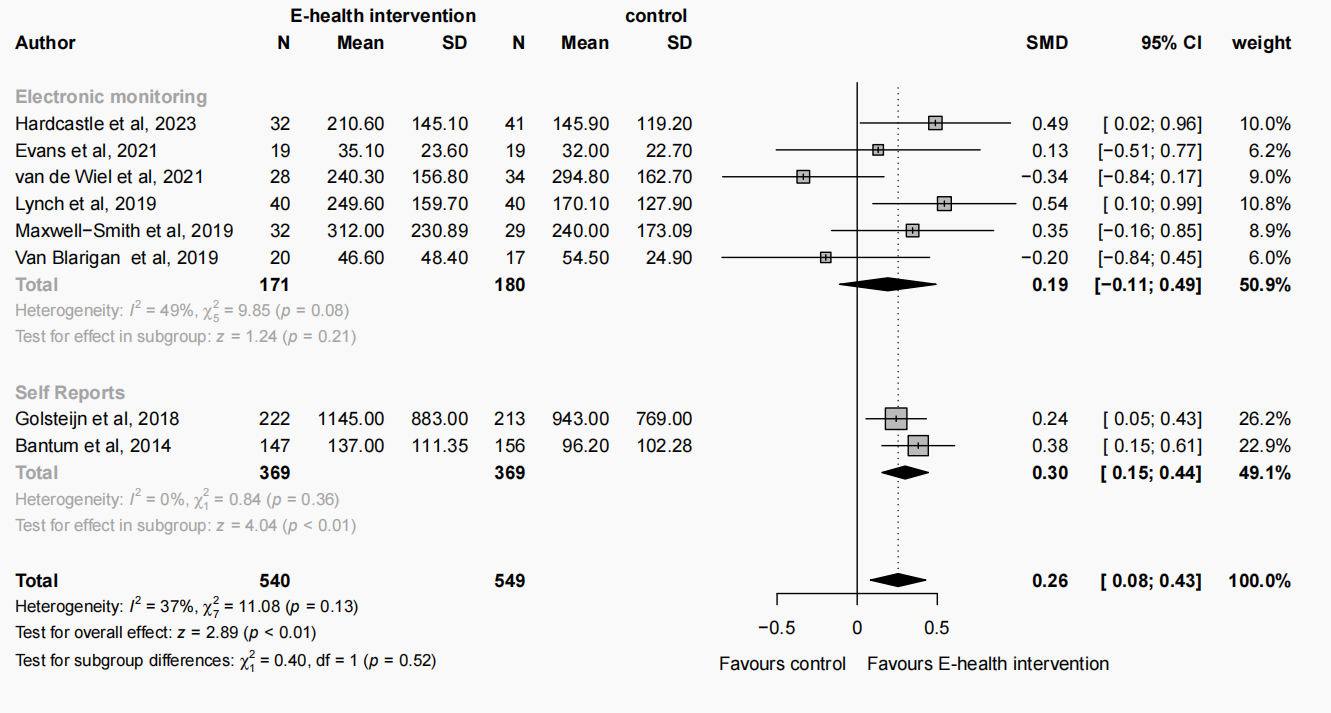


**Figure S21.** Subgroup analysis of MVPA instrument in the effect of E-health interventions on MVPA in cancer survivors (n=8).


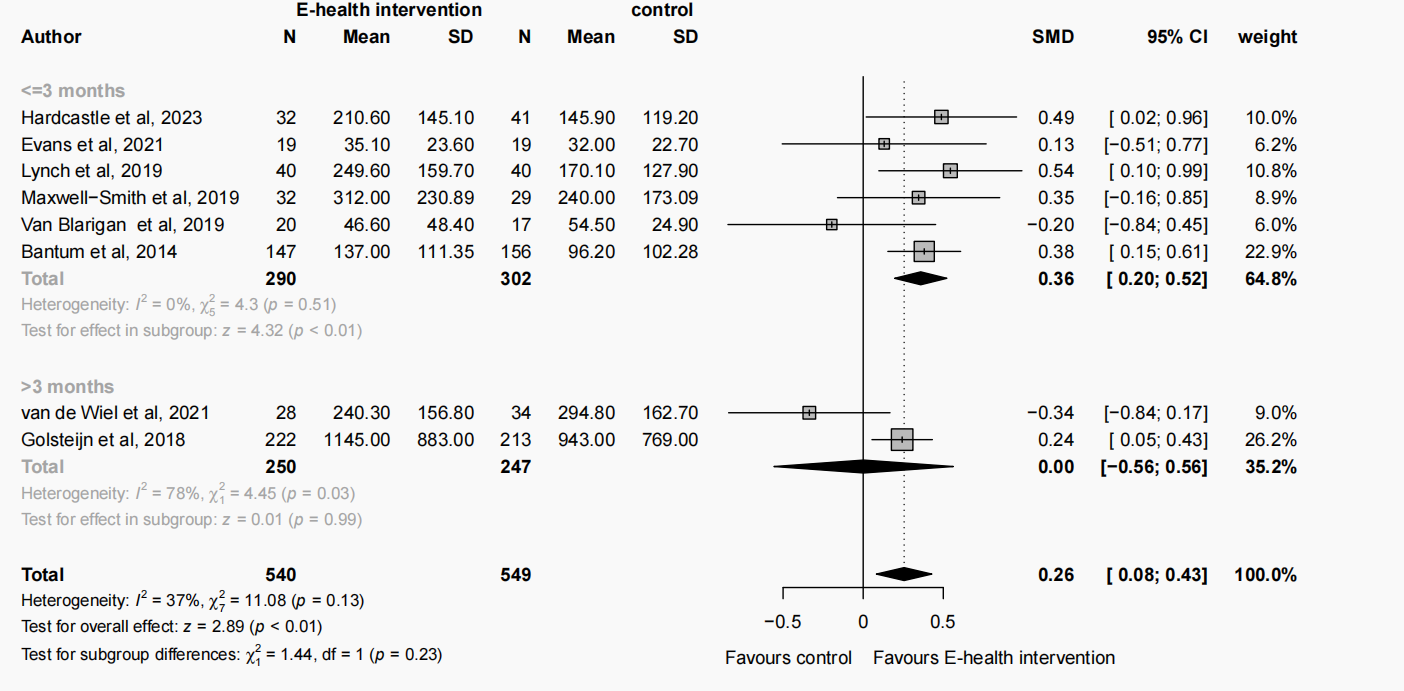


**Figure S22.** Subgroup analysis of E-health intervention duration in the effect of E-health interventions on MVPA in cancer survivors (n=8).


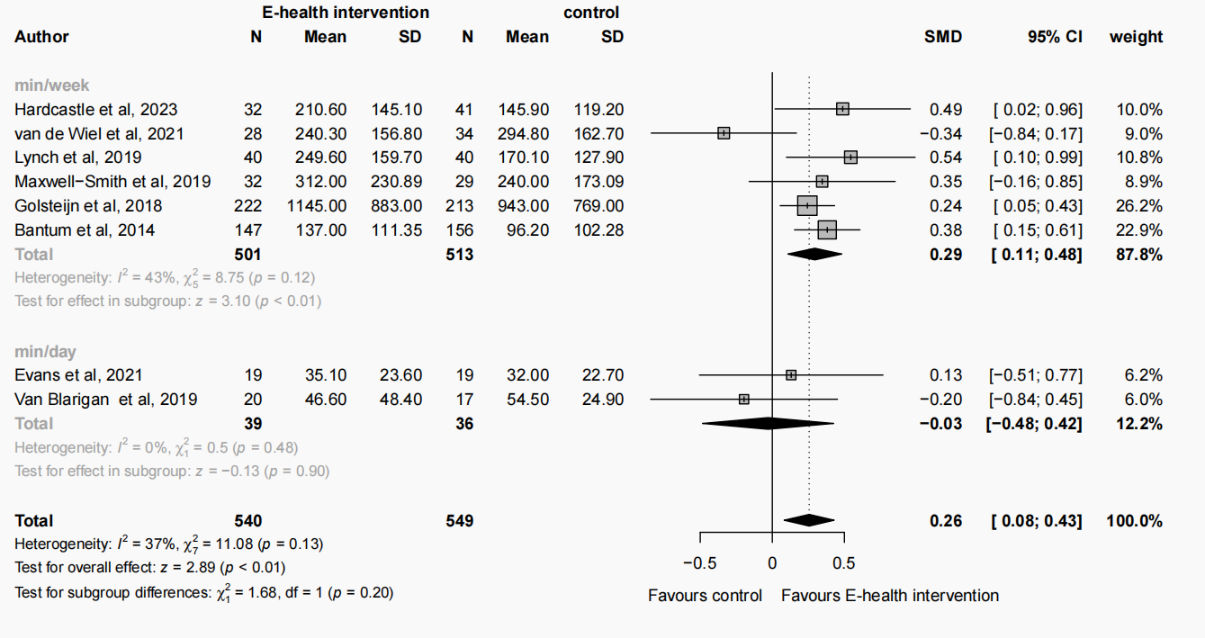


**Figure S23.** Subgroup analysis of MVPA unit in the effect of E-health interventions on MVPA in cancer survivors (n=8).


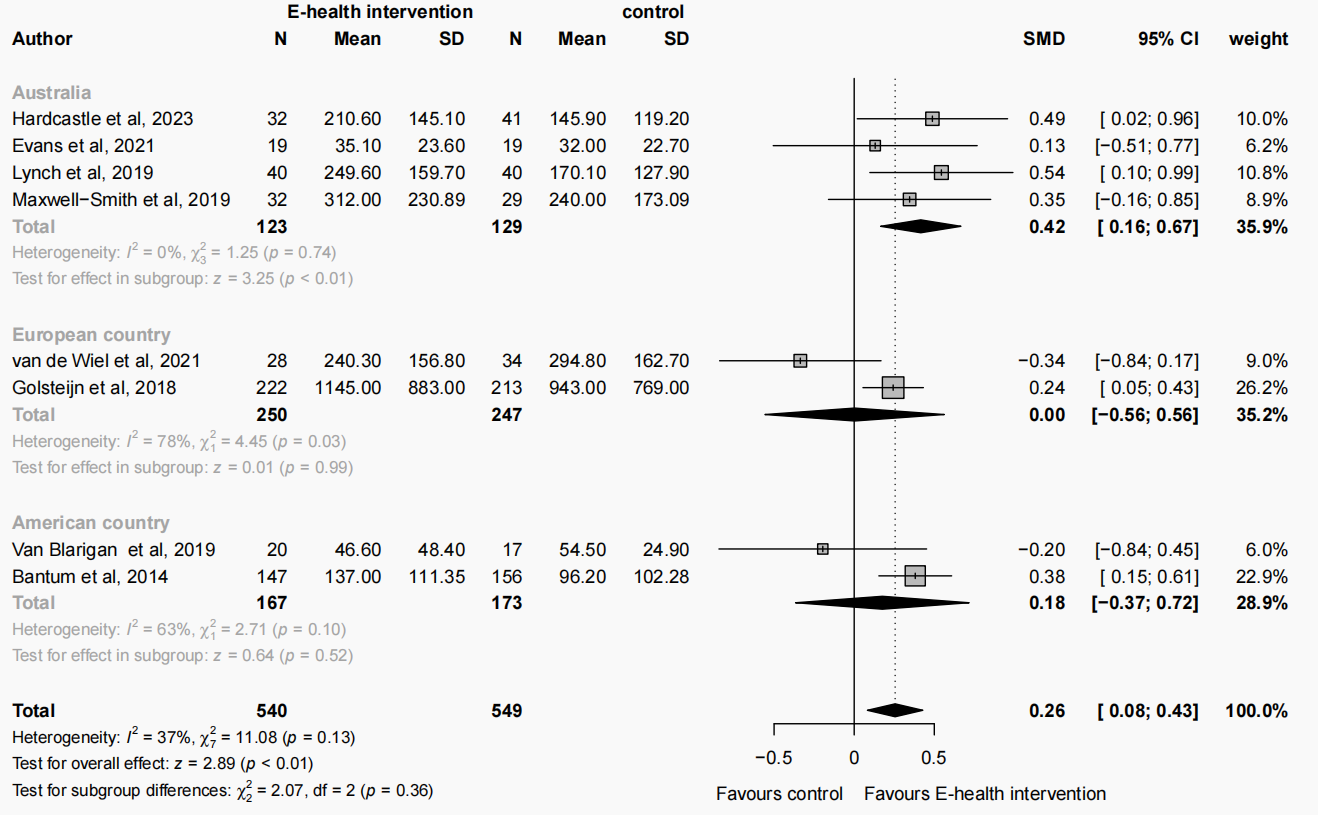


**Figure S24.** Subgroup analysis of country of the included population in the effect of E-health interventions on MVPA in cancer survivors (n=8).


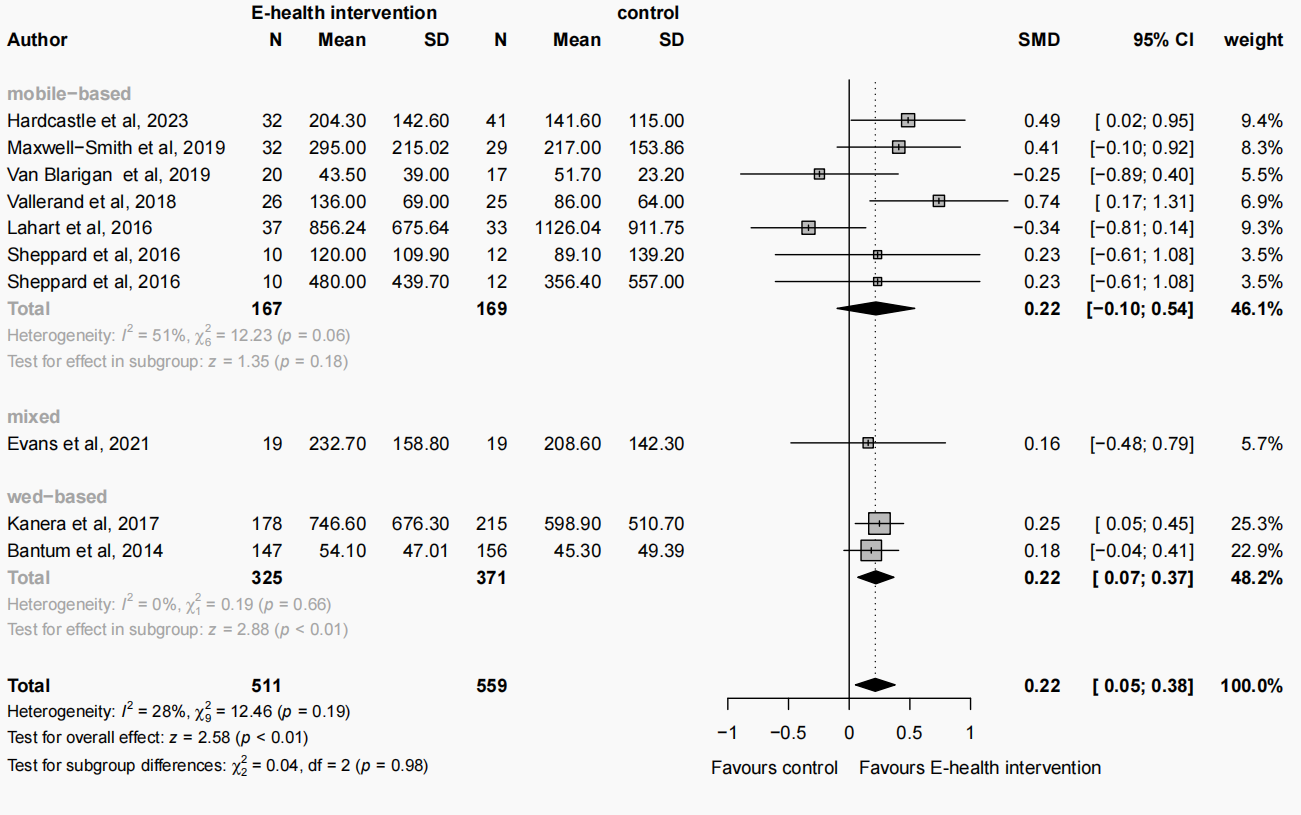


**Figure S25.** Subgroup analysis of E-health intervention method in the effect of E-health interventions on MPA in cancer survivors (n=9).


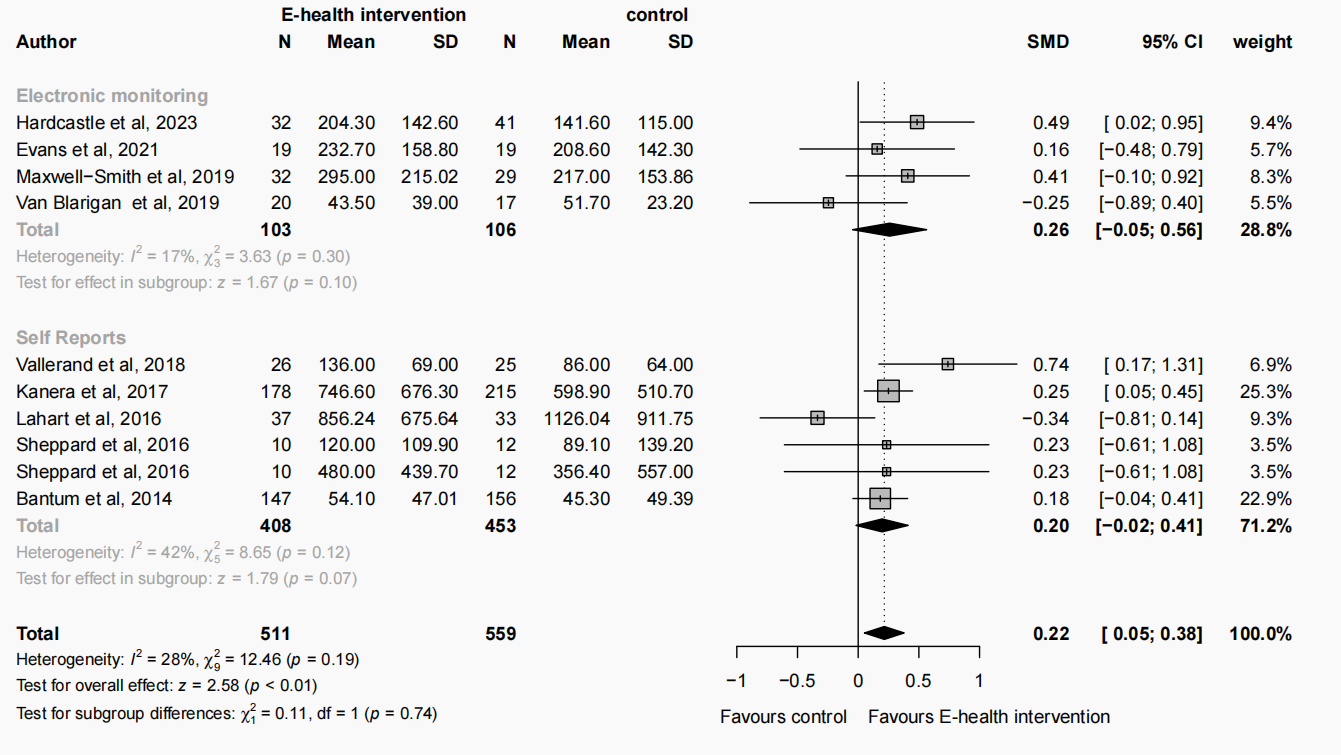


**Figure S26.** Subgroup analysis of MPA instrument in the effect of E-health interventions on MPA in cancer survivors (n=9).


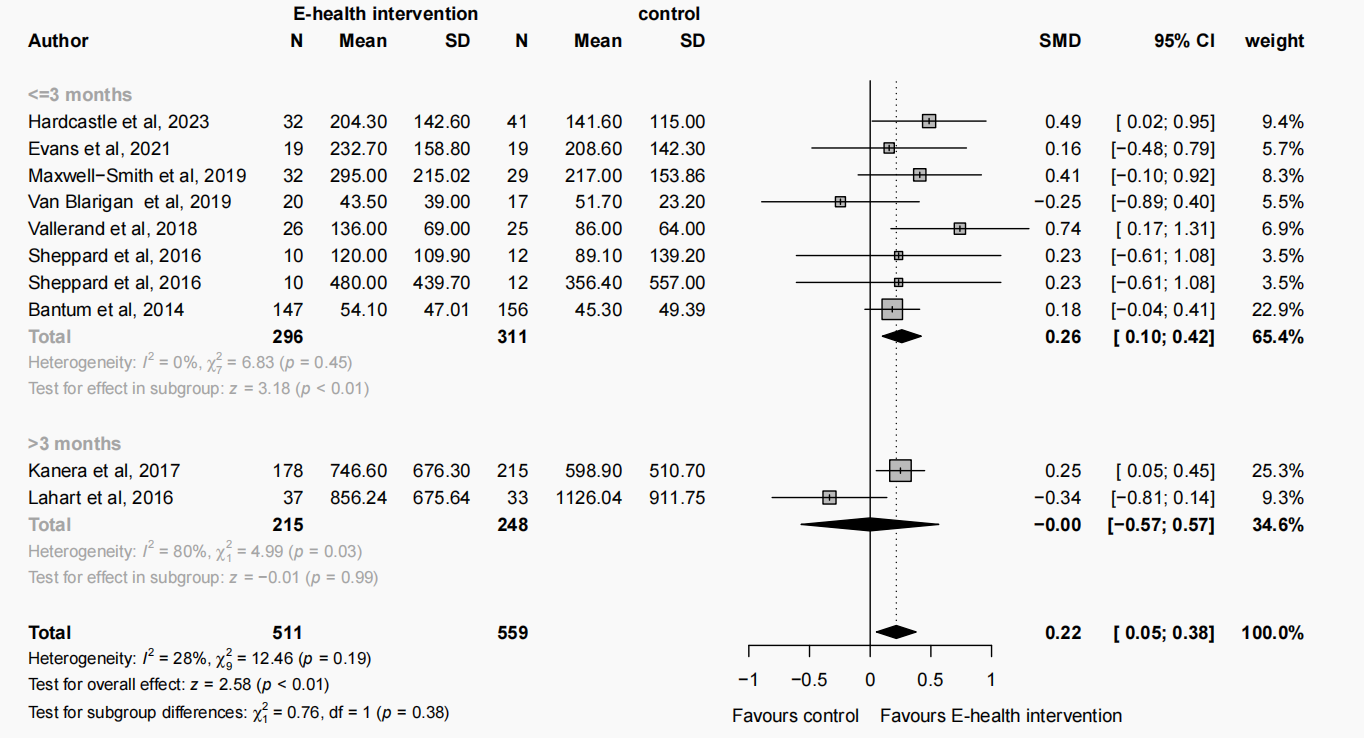


**Figure S27.** Subgroup analysis of E-health intervention duration in the effect of E-health interventions on MPA in cancer survivors (n=9).


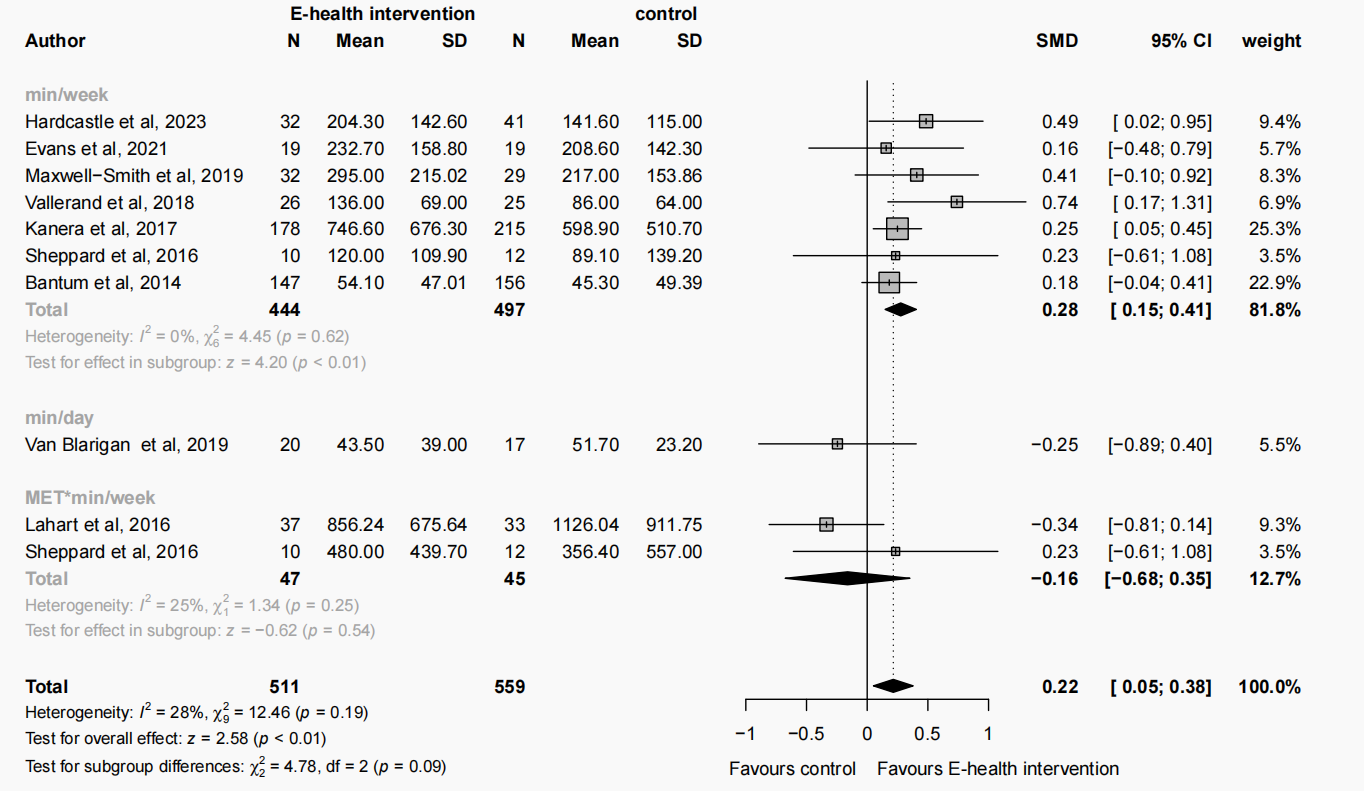


**Figure S28.** Subgroup analysis of MPA unit in the effect of E-health interventions on MPA in cancer survivors (n=9).


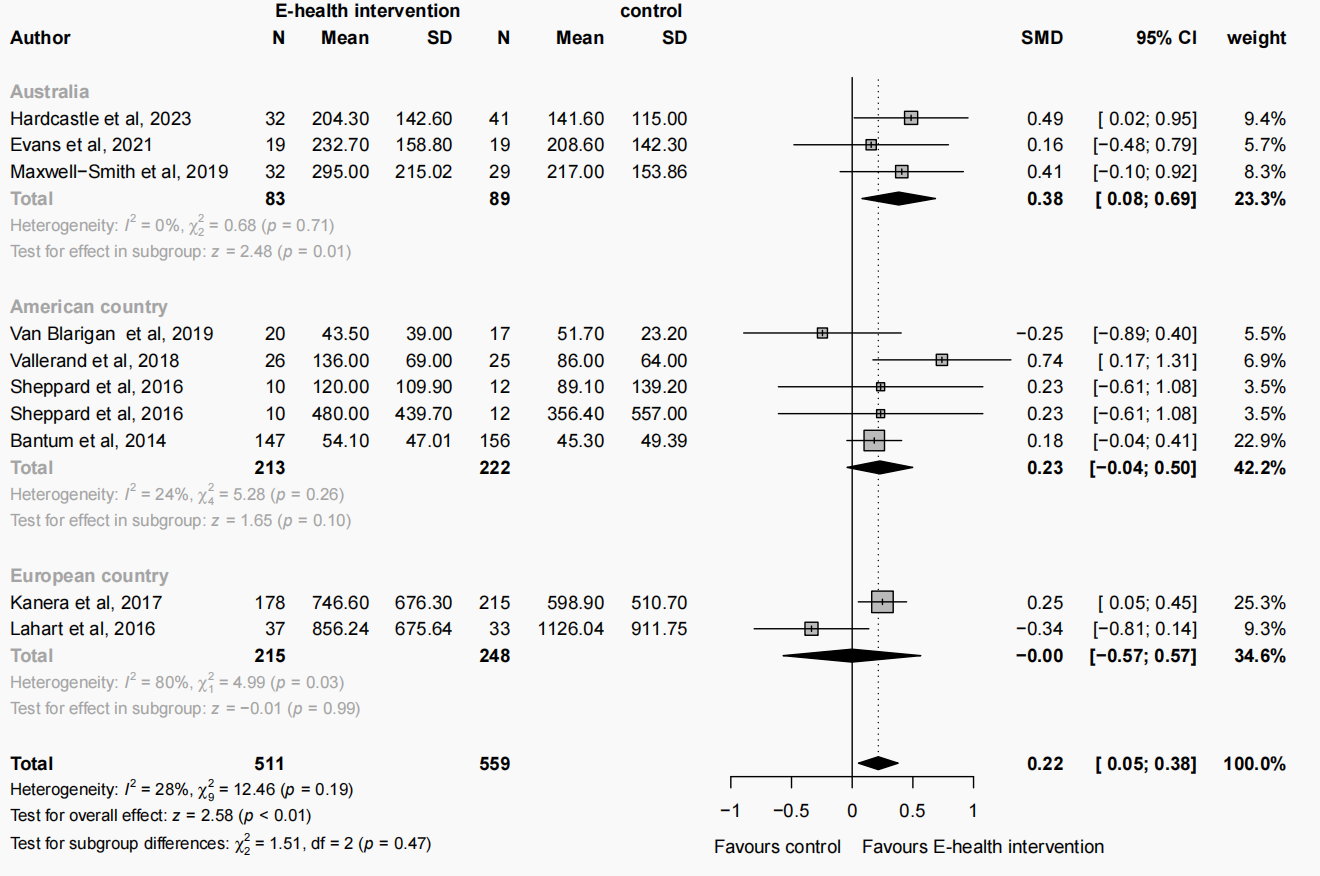


**Figure S29.** Subgroup analysis of country of the included population in the effect of E-health interventions on MPA in cancer survivors (n=9).


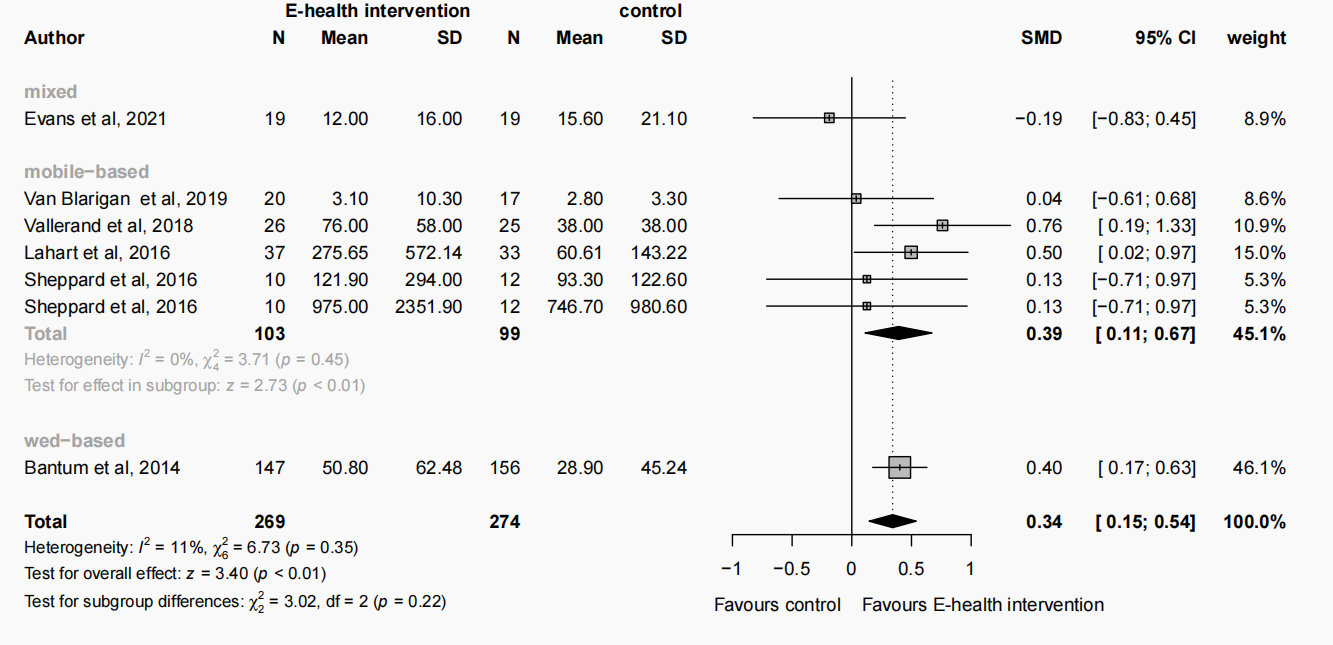


**Figure S30.** Subgroup analysis of E-health intervention method in the effect of E-health interventions on VPA in cancer survivors (n= 6).


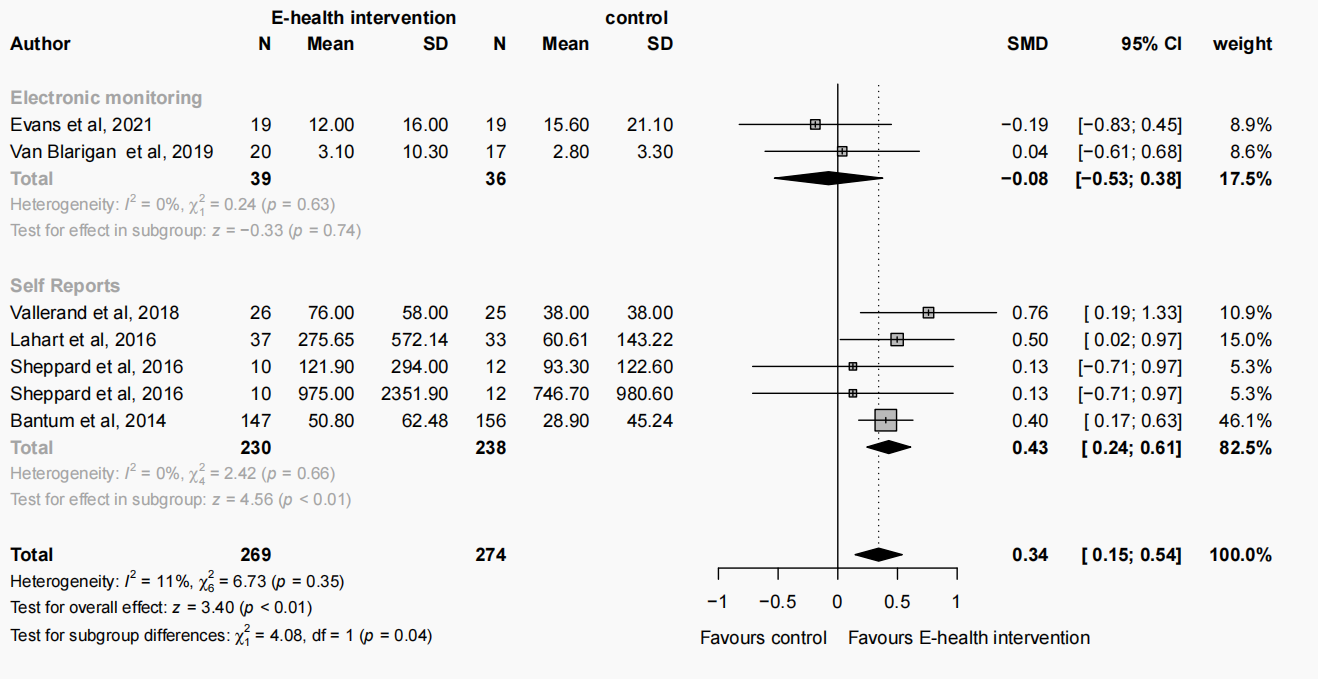


**Figure S31.** Subgroup analysis of VPA instrument in the effect of E-health interventions on VPA in cancer survivors (n= 6).


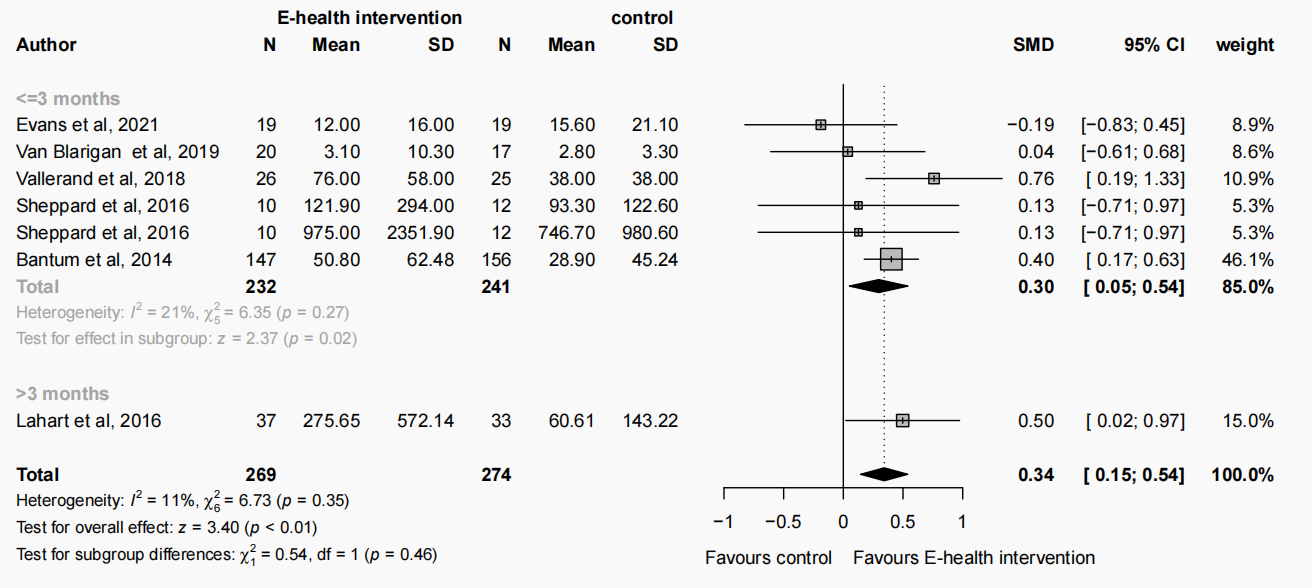


**Figure S32.** Subgroup analysis of E-health intervention duration in the effect of E-health interventions on VPA in cancer survivors (n= 6).


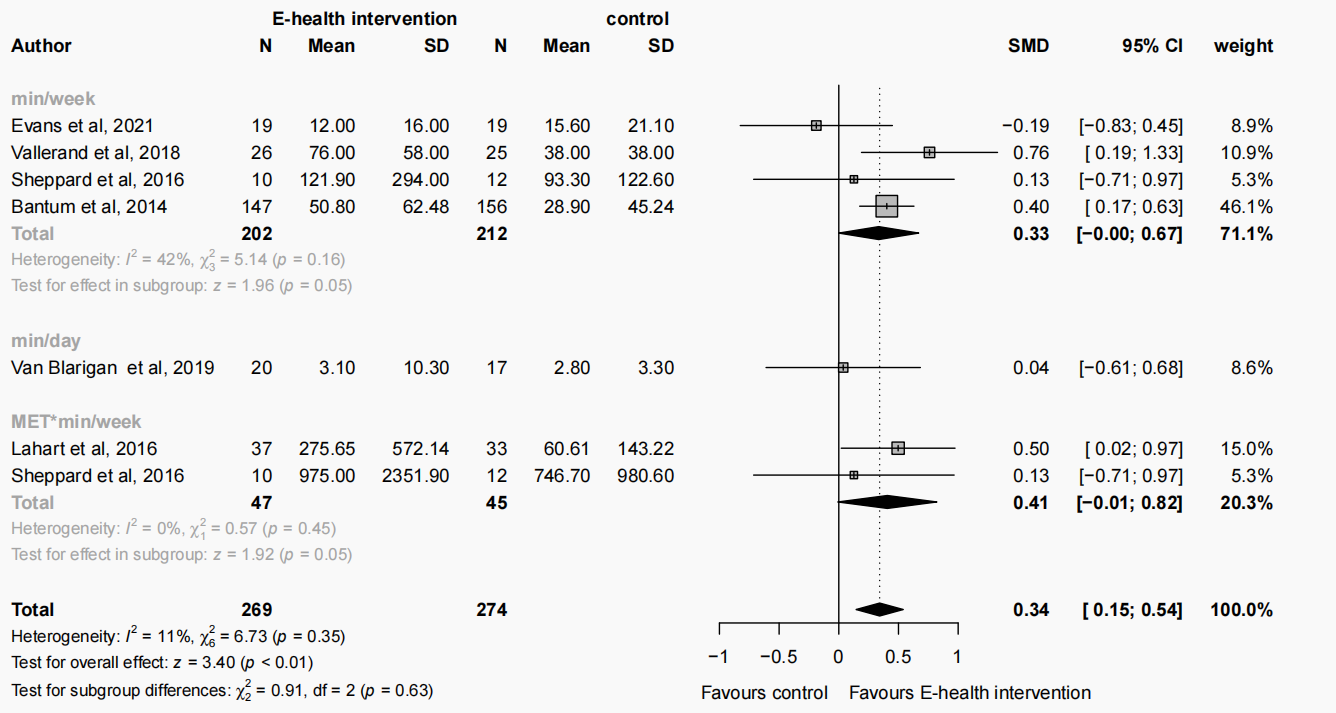


**Figure S33.** Subgroup analysis of VPA unit in the effect of E-health interventions on VPA in cancer survivors (n= 6).


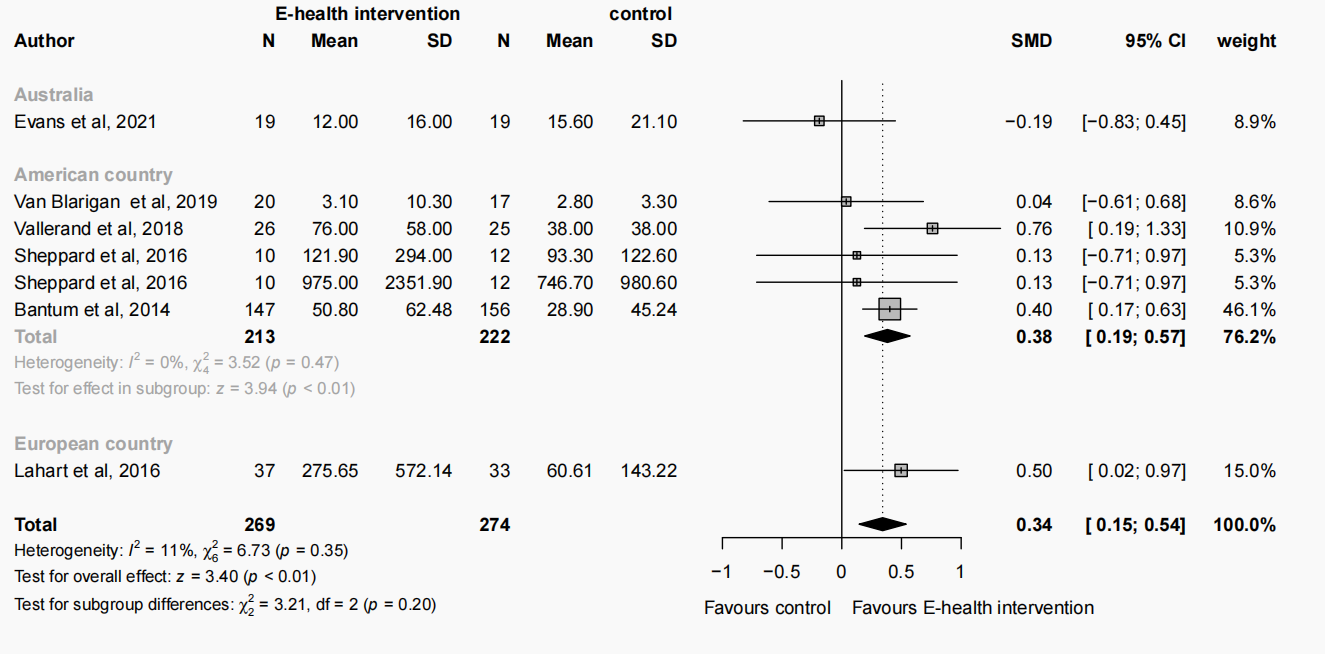


**Figure S34.** Subgroup analysis of country of the included population in the effect of E-health interventions on VPA in cancer survivors (n= 6)
